# The relationship between environmental context and attentional engagement in podcast listening experiences

**DOI:** 10.3389/fpsyg.2022.1074320

**Published:** 2023-01-16

**Authors:** Jay Harrison, Alan W. Archer-Boyd, Jon Francombe, Chris Pike, Damian T. Murphy

**Affiliations:** ^1^AudioLab, School of Physics, Engineering and Technology, University of York, York, United Kingdom; ^2^BBC Research and Development, London, United Kingdom

**Keywords:** attentional engagement, environmental context, mobile audio listening, object–based media, personalized media, personal listening spaces, podcast studies

## Abstract

**Introduction:**

Previous research has shown that podcasts are most frequently consumed using mobile listening devices across a wide variety of environmental, situational, and social contexts. To date, no studies have investigated how an individual's environmental context might influence their attentional engagement in podcast listening experiences. Improving understanding of the contexts in which episodes of listening take place, and how they might affect listener engagement, could be highly valuable to researchers and producers working in the fields of object-based and personalized media.

**Methods:**

An online questionnaire on listening habits and behaviors was distributed to a sample of 264 podcast listeners. An exploratory factor analysis was run to identify factors of environmental context that influence attentional engagement in podcast listening experiences. Five aspects of podcast listening engagement were also defined and measured across the sample.

**Results:**

The exploratory factor analysis revealed five factors of environmental context labeled as: outdoors, indoors & at home, evenings, soundscape & at work, and exercise. The aspects of podcast listening engagement provided a comprehensive quantitative account of contemporary podcast listening experiences.

**Discussion:**

The results presented support the hypothesis that elements of a listener's environmental context can influence their attentional engagement in podcast listening experiences. The soundscape & at work factor suggests that some listeners actively choose to consume podcasts to mask disturbing stimuli in their surrounding soundscape. Further analysis suggested that the proposed factors of environmental context were positively correlated with the measured aspects of podcast listening engagement. The results are highly pertinent to the fields of podcast studies, mobile listening experiences, and personalized media, and provide a basis for researchers seeking to explore how other forms of listening context might influence attentional engagement.

## 1. Introduction

Podcasts are audio recordings that are downloaded or streamed by listeners and most frequently consumed using portable listening devices such as smartphones and tablets (Edison Research, 2019). In recent years the popularity of podcasts has risen sharply with Edison Research and Triton Digital (2022) finding that 41% of participants surveyed in 2021 listened to at least one podcast in the last month, compared to 24% in 2017.

Markman (2015) and Berry (2016) charted the evolution of podcasting as a medium and highlighted its similarities with radio, citing commonalities in production practices, the cultivation of parasocial relationships between host and listener, and the desire to recreate a feeling of “liveness” through social media and other forms of transmedia engagement (Edmond, 2015), despite the “time-shifted” nature of podcast consumption. Despite the advent of on-demand radio, available alongside podcasts through streaming platforms such as BBC Sounds (Berry, 2020), radio is still predominately a linear format, mainly consumed in the car, home, and workplace (Consortium, 2022). In contrast, podcasts are consumed across a wide variety of different environmental (Chan-Olmsted and Wang, 2020), situational (Nyre, 2015), and social contexts (Perks and Turner, 2019), in some cases fluidly traversing multiple changes of context over the course of a single ubiquitous listening experience (Morris and Patterson, 2015).

Spinelli and Dann (2019, p. 118) characterized podcasts as having entered the “repertoire of media used for urban personal listening,” wherein portable listening devices are used with headphones to create isolated personal listening spaces within the wider public space in which the listener is located. Similarly, Bull (2010, p. 56) describes the practice of listeners using portable listening devices and headphones to construct their "very own auditory bubble" within the wider public soundscape. However, the extent to which the listener is able to isolate themselves from the surrounding environmental soundscape is mediated by the level of occlusion provided by the monitoring device used to listen. As such, listeners who consume podcasts using speakers or acoustically transparent headphones are more likely to find their attention is split between the podcast and their environmental context.

Podcasting has traditionally been considered a secondary medium that is often consumed in parallel alongside additional activities competing for the listener's attention (Morris and Patterson, 2015). However, a 2019 study found that 70% of podcast consumers had experience of listening to podcasts without simultaneously engaging in any additional activities (Edison Research, 2019). Chan-Olmsted and Wang (2020) argued that podcasting has now matured into its own distinct medium, separate to radio, and is consumed in different settings to fulfill different listening gratifications. Furthermore, Chan-Olmsted and Wang (2020) found podcast consumption at home tended to be more active and instrumental (Rubin, 1984), being positively associated with information seeking and negatively associated with listening as a form of escapism/pastime. Consumption out of the home, on the other hand, was found to be more ritualized (Rubin, 1984), positively associated with escapism/pastime and negatively associated with information seeking. However, in a study that explored the situational fit of music, radio, and podcasting in urban headphone listening experiences, Nyre (2015) found that podcasts were especially popular amongst “self-curative pedestrian headphone listeners.”

Podcast listening ranks amongst the top media activities for holding audience attention (Insights, 2020). Despite this, however, studies have suggested that as with other forms of audio-based media (Greasley and Lamont, 2011), listeners exhibit different levels of engagement as podcast consumers (Gabriel Tassinari et al., 2020). Existing research has predominately focused on audience engagement with the podcast medium as a function of brand connection (Gabriel Tassinari et al., 2020), social engagement (Tobin and Guadagno, 2022), parasocial relationships (Schlütz and Hedder, 2021), and the amount of listening time (Li et al., 2020), with highly engaged participants characterized as those who make regular financial contributions, develop strong parasocial relationships with podcast hosts, or pass a given threshold of regular listening. Tobin and Guadagno (2022) conducted a study exploring the motivations and outcomes of why people listen to podcasts, in which they outlined five aspects of podcast listening metrics that constituted different ways of engaging with podcasts. These consisted of the amount of time spent listening, the settings in which episodes of listening take place, the editorial format of the podcast, the device(s) used to listen, and social aspects of listening including social and parasocial engagement. García-Marín (2020) conducted qualitative research based on semi-structured interviews with listeners, podcasters, and pioneers in the medium, identifying 13 factors that determine engagement in podcasting. The factors were categorized into three groups of medium-centered, user-centered, and podcast-centered engagement.

Busselle and Bilandzic (2009) developed a scale designed to measure narrative engagement in film and television viewing experiences. Four dimensions of experiential engagement in narratives were defined including narrative understanding, attentional focus, emotional engagement, and narrative presence. Within the dimension of attentional focus, a truly engaged viewer was defined as one who is unaware of their focused attention, up until the point at which their attention drifts and they are required to refocus (Busselle and Bilandzic, 2009, p. 341). When an individual reaches this level of complete attentional focus on an activity they are described as experiencing *flow* with the activity. *Flow* is defined as a state where the individual's attention is fully focused on an activity, paired with, “a loss of conscious awareness of oneself and one's surroundings” (Busselle and Bilandzic, 2009, p. 324). It is this type of attentional engagement, applied in the context of podcast listening experiences, that represents the primary focus of the research conducted in this present study.

Hartmeyer et al. (2017) and Song et al. (2021) conducted studies in the field of auditory neuroscience that used the term *attentional engagement* to refer to a mediating factor in individuals' performance in route planning tasks and narrative comprehension, respectively, whereby one's attentional state fluctuates between different levels of focus on an external task or stimulus. Kaya and Elhilali (2017) conducted a review of studies that model auditory attention. The review found that models of auditory attention can generally be classified as being based around either bottom-up or top-down attention processing. Bottom-up attention occurs in response to external stimuli in the environment that capture the listener's attention, while top-down attention is related to goal oriented attention where an individual actively focuses their attention in order to carry out a pre-planned task or activity. Further research by Berman et al. (2008), Linnell et al. (2013), and White and Shah (2019) has also suggested that an individual's cognitive and attentional processes can be influenced by the nature of stimuli in their surrounding physical environment.

Gaining an understanding of how environmental context may influence attentional engagement could be highly pertinent to research in the fields of object-based media (Armstrong et al., 2014) and related media personalisation disciplines. Gradinar et al. (2015) presented a study on the use of perceptive media in the production of adaptive storytelling experiences that highlighted the weather, temperature, and time of day as factors of a listener's environmental context that could create a deeper level of personalisation, potentially leading to higher attentional engagement. The continued growth in interest around object-based media (OFCOM, 2021) has now given rise to public trials of adaptive experiences that are perceptive to audience context. In 2022 BBC Research & Development released the Adaptive podcasting player app and web editing tool, which enable the production of audio experiences that are personalized according to data from the listener's device and elements of their surrounding environmental context (Stagg, 2022).

### 1.1. Research aims

This study aims to identify and investigate how different factors of environmental context might relate to listeners' attentional engagement when consuming podcasts using a smartphone. Furthermore, it aims to quantitatively map out how listeners consume podcasts across several *aspects of podcast listening engagement*, and explore how these aspects relate to the proposed *factors of environmental context*. Results from this study may also be of relevance to future work in related research fields, therefore it is also a stated aim of this study to evaluate potential implications of this work in the context of podcast studies, media personalisation, and attentional processing research.

The first research question asks how different factors of environmental context relate to listeners' attentional engagement when listening to podcasts. This question was primarily concerned with the identification of different factors which would then permit further measurement, analysis, and hypothesis testing. It is hypothesized that questionnaire items will group together under simple structure criteria (Thurstone, 1947) to form factors that meaningfully define elements of environmental context that influence listeners' attentional engagement when consuming podcasts using a mobile device [H1].

The second research question asks how podcast consumers engage with podcasts across several *aspects of podcast listening engagement* and how the aspects quantitatively relate to one another. The *aspects of podcast listening engagement* investigated are the amount of listening, the locations in which episodes of listening take place, the monitoring devices used to listen, the multitasking activities engaged in while listening, and the methods used to discover podcasts. It is hypothesized there will be positive correlations observed amongst the *aspects of podcast listening engagement* [H2].

The third research question asks how the proposed *factors of environmental context* relate to the measured *aspects of podcast listening engagement*. For this question it is also hypothesized that there will be positive correlations amongst the *environmental context factor scores* and *aspects of podcast listening engagement* [H3].

## 2. Materials and methods

### 2.1. Participants

A sample of 264 people aged 18–66+ (18–25 = 13.6%, 26–35 = 42.8%, 36–45 = 18.6%, 46–55 = 17.0%, 56–65 = 7.2%, 66+ = 0.8%) completed an online questionnaire. The majority of the sample was male (51.9%), 43.2% were female, 3.8% non-binary/third gender, and 1.1% preferred not to answer. Of the 264 participants, 134 (50.8%) resided in the United Kingdom, 51 (19.3%) in the rest of Europe, 47 (17.8%) in the United States of America, and 32 (12.1%) in the rest of the World. Participants were recruited online *via* various methods including newsletters, social media posts (e.g., Facebook and Twitter), research group networks, University department mail-outs, podcasts, and word of mouth. To take part in the study participants first had to confirm that they had experience of listening to podcasts using a smartphone, in this sense the sample is representative of the non-zero podcast listening population. Responses were collected between the 29th of November 2021 and the 9th of February 2022. Participant involvement in the study was exclusively on a voluntary basis.

### 2.2. Measures

Participants first provided their age, gender, and country of residence. To answer the second and third research questions, which asked how aspects of podcast listening engagement relate to one another and to the proposed *factors of environmental context*, participants were first asked a series of quantitative and qualitative questions concerning their podcast listening behaviors and habits while using a smartphone.

Participants first selected how much time they spent listening on an average weekday, and then an average weekend day, from a list of nine options ranging from 0 min to more than 10 h. They were then asked to select all of the locations in which they had listened to a podcast with a smartphone from a list of eight options. An *Other not listed (please specify)* option was also included with a free text response box to allow participants to register additional locations that were not included in the default survey options. Participants were also asked to select the location in which they most often listened from a list that was populated by their answers to the first question.

To collect data on the monitoring devices listeners use to consume podcasts, participants selected all of the headphone- and loudspeaker-based devices they had used with a smartphone to listen, from a list of eight options. An *Other not listed (please specify)* option was also included with a free text response box. Participants were again asked to select the monitoring device they most often used from a list that was populated by their answers to the first question. Participants were then asked to select all of the activities they had engaged in while listening to podcasts with a smartphone from a list of 12 options. An *Other not listed (please specify)* option was also included with a free text response box. Participants were also asked to select the activity they most often used from list that was populated by their answers to the first question.

Finally, participants were asked to select all of the methods they had used to discover podcasts from a list of 8 options. An *Other not listed (please specify)* option was also included with a free text response box. Participants were again asked to select the method they most often used from a list that was populated by their answers to the first question.

This data was collected as a quantitative measure of different aspects of participants' podcast listening engagement, adapted from the aspects of podcast listening engagement metrics originally presented by García-Marín (2020) and Tobin and Guadagno (2022) in order to reflect the present study's interest in environmental context. The data collected in this part of the study was analyzed to calculate the total number of responses provided by individual participants for each *aspect of podcast listening engagement*. For example, the total number of unique methods that a participant reported using to discover podcasts would provide a measure of their podcast discovery engagement level. This data was then used to answer the second and third research questions.

**Figure 1:**
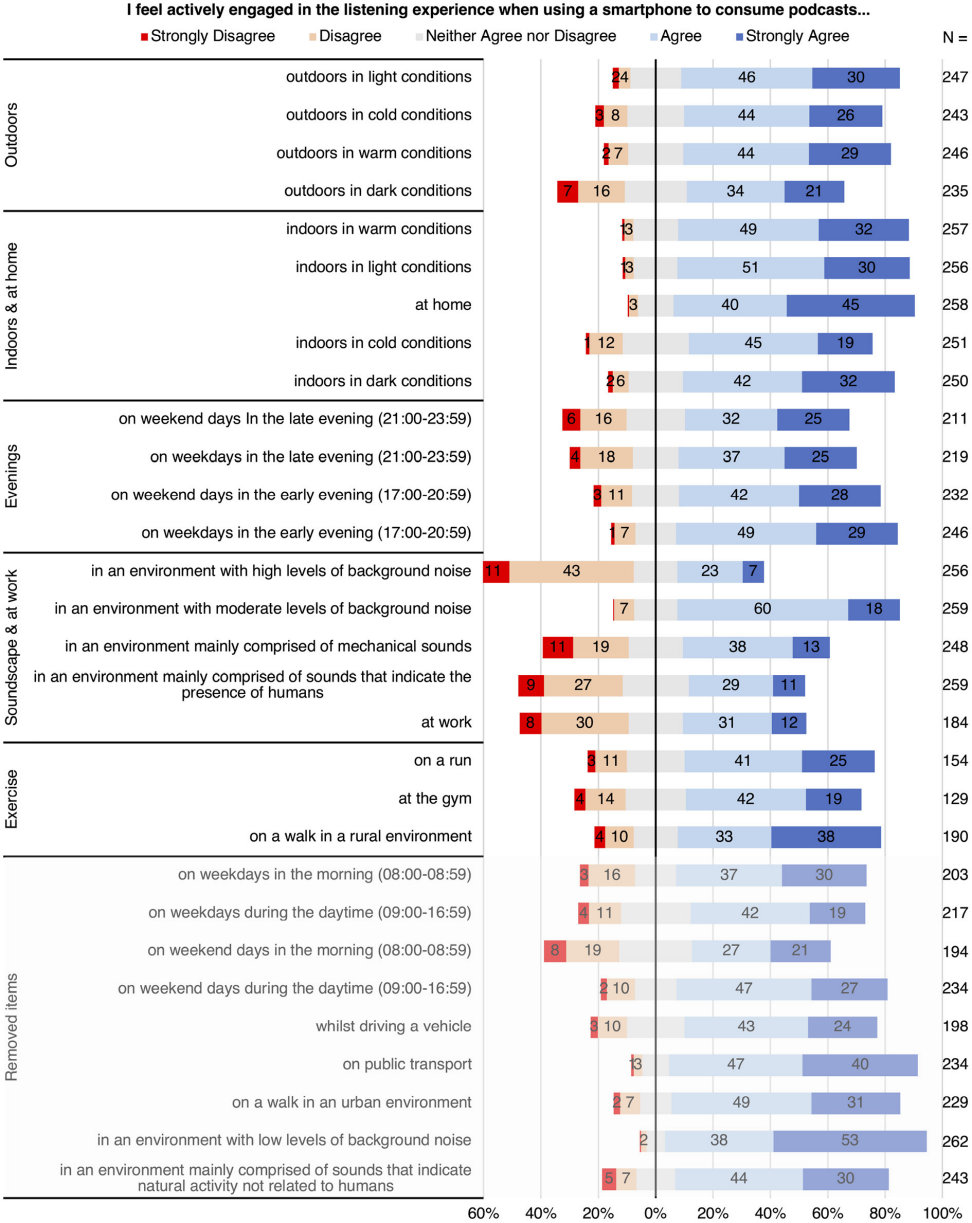
Attentional engagement as a function of environmental context in podcast listening experiences scale with the factors of environmental context produced by the EFA.

To answer the first and third research questions, a 30-item *Attentional engagement as a function of environmental context in podcast listening experiences* scale (Figure 1) was constructed based on previous literature concerning influencing factors of environmental context on emotional response to music (Susino, 2021), choice, and devices used in everyday music listening (Krause et al., 2015). Participants were asked to what extent each item was representative of their observed attitudinal experience on a discrete 5-point Likert scale (1 = strongly disagree to 5 = strongly agree). An “NA” option was also included on each item, allowing participants to indicate if insufficient contextual listening experience prevented them from providing a response. The 30 items were revised from a larger collection through the removal of unclear and repetitive items and further review by two independent music psychology experts and a podcast industry professional. All items began with the statement “I feel actively engaged in the listening experience when using a smartphone to consume podcasts...” with the term “actively engaged” defined in the survey as, “an attentional state where the listener is fully focused on the listening experience.”

Two optional free text questions asked participants to first describe how different factors of environmental context influenced their level of engagement when listening to podcasts using a smartphone, and then how different factors of environmental context influenced their preference for listening to specific types of podcasts using a smartphone.

The survey also included several ancillary questions intended to gather data that would inform future research in the associated Ph.D. project, the results of which are not included in this paper. The median time participants took to complete the survey was 10 min and 9 s. There were several outliers who registered longer elapsed completion times as they completed the survey over multiple sittings. A full copy of the survey instrument and survey logic is provided in Supplementary Figure 1.

### 2.3. Procedure

The University of York Electronic Engineering department ethics committee approved this study (approval number Harrison101121). Participants accessed the study online using a link to the participant information sheet. Participants were required to provide their consent prior to viewing and completing the questionnaire using the Qualtrics online survey platform. None of the participants who took part received any remuneration for their participation in the study.

## 3. Results

### 3.1. Factors of environmental context

To answer the first research question an exploratory factor analysis (EFA) was conducted to investigate the presence of underlying latent variables in the 30 item scale used in the online questionnaire, measuring factors of environmental context that influence listeners' attentional engagement in podcast listening experiences. The sample comprised 264 participants and included missing data due to *not applicable* item responses. 13.6% of responses across all items were classified as missing data, ranging from 51.1 to 0.8% for individual items. Missing data frequencies for each item can be attained from the sample size data reported in Figure 1.

Sampling adequacy tests were initially conducted using pairwise deletion, resulting in an overall Kaiser–Meyer–Olkin (KMO) sampling adequacy measure of 0.791 and individual item measures all >0.633. All items were consequently classified between “Mediocre” to “Meritorious” as interpreted by Kaiser (1974). Bartlett's test of sphericity was significant at *p* < 0.05, suggesting it was likely the data could be factorized. Mardia's Multivariate Normality Test indicated that the data was not normally distributed (skew = 5963.36, kurtosis = 3.33) at *p* < 0.001.

The Multiple Imputation Factor Analysis (MIFA) R package (Nassiri et al., 2021) was used to impute missing data and indicate factor retention threshold criteria for further analysis. The incomplete dataset was imputed M = 30 times (Nassiri et al., 2018) using the fully conditional specification (FCS) (van Buuren, 2007) with the predictive mean matching (PMM) method. Confidence intervals for the cumulative proportion of explained variance were derived from principal component eigenvalue decomposition using Rubin's rules (Rubin, 2004) and the average of proportions of explained variance over all imputed datasets. The EFA was then performed on an averaged estimated covariance matrix produced by the MIFA package with a Principal Axis Factoring (PAF) extraction method (Gibson et al., 2020) using the *fa* function (Revelle, 2022). The disparity observed between the MIFA confidence intervals used to indicate factor retention threshold criteria and the consequent cumulative proportion of explained variance produced by the EFA and shown in Table 1 can be attributed to this distinction. However, this methodology is consistent with guidance describing its implementation provided by Busch and Nassiri (2021) and was further confirmed in email correspondence from Nassiri (V Nassiri 2022, personal communication, 4 April).

**Table 1:** Factor loadings for the EFA with direct quartimin rotation of the retained “environment context attentional engagement podcast listening” items.

	**Factor**				
**Item**	**1**	**2**	**3**	**4**	**5**
Outdoors in light conditions	0.806				
Outdoors in cold conditions	0.753				
Outdoors in warm conditions	0.735				
Outdoors in dark conditions	0.610				
Indoors in warm conditions		0.924			
Indoors in light conditions		0.826			
At home		0.570			
Indoors in cold conditions		0.477			
Indoors in dark conditions		0.406			
On weekend days in the late evening (21:00–23:59)			0.859		
On weekdays in the late evening (21:00–23:59)			0.753		
On weekend days in the early evening (17:00–20:59)			0.710		
On weekdays in the early evening (17:00–20:59)			0.523		
In an environment with high levels of background noise				0.784	
In an environment with moderate levels of background noise				0.558	
In an environment mainly comprised of mechanical sounds				0.548	
In an environment mainly comprised of sounds that indicate the presence of humans				0.548	
At work				0.401	
On a run					0.970
At the gym					0.780
On a walk in a rural environment					0.423
SS loadings	2.696	2.557	2.373	1.963	1.895
Proportion of variance (%)	12.837	12.174	11.301	9.348	9.023
Proportion explained (%)	23.476	22.263	20.667	17.094	16.500

Analysis conducted using MIFA dataset (*N* = 264). Primary loadings <0.4, alternative loadings <0.3, and alternative loadings with a difference of <0.2 suppressed. The five factors were labeled by the first author as *outdoors, indoors & at home, evenings, soundscape & at work*, and *exercise*.

Review of the correlation matrix showed that all items had at least one correlation with a coefficient >0.30, except for “*whilst driving a vehicle.”* This item was removed, and the EFA rerun with the remaining 29 items (Hair et al., 2010). As the dataset was not normally distributed a bootstrap, as opposed to Fieller, confidence interval was employed to determine factor retention criteria using the MIFA function (Nassiri et al., 2018). As the first five factors were found to explain at least an estimated 5.0% of the total variance individually and 55.20% cumulatively, with bootstrap confidence intervals of 0.547 and 0.606, a proposed five-factor solution was considered for retention (Nassiri et al., 2018). In order to support this proposed solution, separate parallel analysis, and visual inspection of scree plots were conducted on the original incomplete 29 items using pairwise deletion to address missing data (Goretzko et al., 2021). This initially suggested that six factors (or seven principal components) should be retained; however, when further analysis using a forced six-factor solution was carried out, the sixth factor failed to satisfy the ≥3 primary loadings per factor criterion outlined by Howard (2016). Therefore, a five-factor solution was selected for retention and further analysis.

The EFA was rerun with a forced five-factor solution. As factors could potentially be correlated a direct quartimin rotation method was applied to improve interpretability, providing an equal weighting between correlated and uncorrelated factors (Howard, 2016). Item factor loading criteria were drawn from a systematic review conducted by Howard (2016), with items with primary factor loadings above 0.40 retained, while items with alternative loadings below 0.30 and cross-loadings with a difference of 0.20 or larger were cut. This resulted in the removal of the *weekday morning, weekday daytime, weekend morning, weekend daytime, public transport, urban walk environment, low background noise*, and *human soundscape* items. These removed items are noted in the lower, faded out, section of Figure 1.

MIFA was rerun using the same criteria and again suggested a five-factor solution. This was supported by separate parallel analysis and visual inspection of scree plots performed on the incomplete 21 items using pairwise deletion to address missing data. This also suggested a five-factor (or five principal component) solution and consequently the EFA was rerun on the remaining 21 items using the same criteria. The overall KMO sampling adequacy measure was 0.792, with all individual measures greater than 0.638. All items were classified between “Mediocre” to “Meritorious” as interpreted by Kaiser (1974). The bootstrap confidence intervals for the proportion of explained variance using five factors were 0.634 and 0.690, and the equivalent Fieller's intervals were 0.613 and 0.666. The estimated proportion of explained variance for the first five factors was 0.640.

The rotated solution this produced demonstrated simple structure (Thurstone, 1947) and is shown in Table 1. The interpretation of the data was consistent with the proposed hypothesis and exhibited strong loadings across the five factors. Bivariate correlations between the five factors are shown in Table 2. Items associated with listening *outdoors* loaded on Factor 1, items describing listening *indoors & at home* on Factor 2, items relating to listening in the *evenings* on Factor 3, items pertaining to listening *soundscape & at work* on Factor 4, and listening while engaging in *exercise* on Factor 5. Cumulatively the five factors were able to explain 56.06% of the total variance across all 21 items.

**Table 2:** Pearson's product-moment correlations amongst the five factors produced by the EFA (*N* = 264).

		**Outdoors**	**Indoors & at home**	**Evenings**	**Soundscape & at work**	**Exercise**
Outdoors	PCC	–			
	Sig.	.			
Indoors & at home	PCC	0.457^**^	–		
	Sig.	<0.001	.		
Evenings	PCC	0.207^**^	0.263^**^	–	
	Sig.	<0.001	<0.001	.	
Soundscape & at work	PCC	0.313^**^	0.258^**^	0.238^**^	–
	Sig.	<0.001	<0.001	<0.001	.
Exercise	PCC	0.244^**^	0.205^**^	0.146^*^	0.334^**^	–
	Sig.	<0.001	<0.001	0.018	<0.001	.

^*^Correlation is significant at the 0.05 level (2-tailed). ^**^Correlation is significant at the 0.01 level (2-tailed).

An additional EFA was conducted using identical analysis criteria with pairwise deletion (Goretzko, 2021) and visual inspection of parallel analysis scree plots (Cattell, 1966) to further support the results obtained from the MIFA solution. This produced the same factor loadings with the only exceptions being the *on a walk in a rural environment* item exhibiting a weak cross-loading of 0.489 and 0.330 on the *exercise* and *outdoors* listening factors respectively and Factors 4 and 5 reversing in order of proportion of variance explained due to a difference of 0.81%. Therefore, it can be determined, that as items from the Likert scale grouped together under simple structure criteria to form five *factors of environmental context* that influence attentional engagement, the null hypothesis can be rejected and the alternate hypothesis [H1] accepted.

*Environmental context factor scores* were then computed for each participant using the method described by Busch and Nassiri (2021), providing a measure of participants' attentional engagement for each of the five *factors of environmental context*. An overall mean factor score was also computed for each participant from the five *environmental context factor scores* to provide a cumulative measure of attentional engagement as a function of the *factors of environmental context* shown in Table 1.

### 3.2. Relationships between aspects of podcast listening engagement

To answer the second and third research questions, which collectively asked how listeners engage with podcasts across several *aspects of podcast listening engagement*, how these aspects relate to one another, and how they relate to the proposed *factors of environmental context*, participants were asked a series of questions intended as a quantitative measure of their podcast listening engagement and listening behaviors. The following section presents an outline of this data, followed by the results of correlation tests that were carried out to identify potential relationships between the aspects.

#### 3.2.1. Amount of listening time

Response frequencies for the questions measuring the amount of time participants spend listening are shown in Table 3. The median category for the amount of listening time on an average weekday amongst all participants was 60–120 min, accounting for 26.14% of all recorded responses. The median category for an average weekend day was 30–60 min, which accounted for 28.03% of all responses. Additionally, there was only one participant who reported not listening on weekdays, compared to 20 who reported not listening on weekend days. The Likert scale *not applicable* response results presented in Figure 1 indicate that episodes of listening occurred fairly consistently throughout the week. Early evenings on weekdays was the most popular time to listen with 93.18% of participants reporting having listened during this time, while weekend days in the morning was the least popular with 73.48%.

**Table 3:** Amount of time participants spend listening to podcasts using a smartphone on average weekdays and weekend days (*N* = 264).

	**Average weekday**		**Average weekend day**	
**Listening amount**	**Frequency**	**%**	**Frequency**	**%**
More than 10 h	2	0.76	1	0.38
5–10 h	13	4.92	0	0.00
3–5 h	25	9.47	11	4.17
2–3 h	25	9.47	27	10.23
1–2 h	69	26.14	68	25.76
30–60 min	60	22.73	74	28.03
15–30 min	45	17.05	34	12.88
<15 min	24	9.09	29	10.98
0 min	1	0.38	20	7.58
Total	264	100.00	264	100.00

#### 3.2.2. Listening locations

Response frequencies for the questions concerning locations in which participants consumed podcasts are shown in Table 4. The data presented in the “as a % of location cases” column describes locations in which podcasts are most often consumed as a proportion of the total cases for each location.

**Table 4:** Locations in which participants have consumed (*N* = 264) and most often consume (*N* = 264) podcasts using a smartphone.

	**Locations in which podcasts**			**Locations in which podcasts**		
	**are consumed**			**are most often consumed**		
**Location**	**Frequency**	**%**	**As a % of sample**	**Frequency**	**%**	**As a % of location cases**
At home	245	20.82	92.45	126	47.73	51.43
Traveling on public transport	193	16.40	72.83	21	7.95	10.88
Walking in an urban environment	190	16.14	71.70	48	18.18	17.95
Driving a vehicle	156	13.25	58.87	28	10.61	17.95
Walking in a rural environment	108	9.18	40.75	14	5.30	12.96
On a run	88	7.48	33.21	7	2.65	7.95
At work (private)^***^	72	6.12	27.17	5	1.89	6.94
At the gym	61	5.18	23.02	2	0.76	3.28
At work (public)^***^	43	3.65	16.23	7	2.65	16.28
Other (public)^*^	15	1.27	5.66	5	1.89	33.33
Other (private)^*^	6	0.51	2.26	1	0.38	16.67
Total	1,177	100.00	445.83	264	100.00	–
Listening location groups^**^						
Public locations	698	59.30	264.39	104	39.39	–
Private locations	479	40.70	181.44	160	60.61	–

^*^Computed from *Other not listed (please specify)* responses. ^**^Computed from location responses according to analysis criteria outlined in the results section. ^***^Computed from at work and commuting response data.

Participants' homes were reported as the location in which podcasts were most frequently consumed, with 92.45% of those surveyed having listened at home and 47.73% stating they most often listened at home. The next most popular location was listening while traveling on public transport with 72.83%, followed by listening while walking in an urban environment at 71.70%, however, listening while walking in a rural environment was much lower at 40.75%. Similarly, 18.18% of participants reported most often listening while walking in urban environments, compared to only 5.30% that most often listened while walking in rural environments.

It should also be noted, however, that these results highlight inconsistencies in the data collected by this study. While the data shown in Table 4 indicates that the percentage of participants who listened while walking in an urban and rural environment was 71.70 and 40.75% respectively, the *not applicable* response data provided to the Likert item questions in Figure 1 suggests that 86.75% of participants listened while walking in urban environments, compared to 71.67% that listened while walking in rural environments. The most likely explanation for this discrepancy is an element of self-response bias in the Likert item response data. Despite this, it is still noteworthy that both sets of data are similarly distributed, with listening while walking in an urban environment registering a much higher proportion of cases in both instances.

Further analysis was also conducted to investigate how participants' listening episodes were distributed between private and public spaces. Criteria for listening location group categorization was adapted from a study on the influence of location in everyday experiences of music conducted by Krause et al. (2016). The *private location* group consisted of *at home, at work (private), driving a car*, and *other (private)* responses, while the *public location* group comprised all remaining locations in Table 4. The *other (private)* variable was computed manually *via* a process of categorizing the *Other not listed (please specify)* responses as either private or public according to the free text data provided by participants. The *at work (private)* and *(public)* variables were computed by filtering the *at work* responses according to whether or not the participant commuted to their workplace.

This showed that 59.30% of all locations in which participants listened were public spaces, while the remaining 41.70% were private. However, 60.61% of participants reported most often listening in a private space, compared to 39.39 % who most often listened in public. Further analysis revealed that the vast majority of participants (87.88%) had experience of listening in both private and public locations. 96.21% had experience of listening in at least one private location, while 91.67% reported having listened in at least one public location. This was contrasted by 8.33% of participants who reported only having experience of listening in private locations, and just 3.79% who had only listened in public locations.

#### 3.2.3. Monitoring devices

Response frequencies for the two questions on the monitoring devices used by participants to consume podcasts are shown in Table 5. The data presented in the “as a % of device cases” column describes the monitoring devices most often used to consume podcasts as a proportion of the total cases for each device. The results showed that there was an almost equal split across monitoring devices used by the most participants, between built-in smartphone speakers (55.30%), wired in-ear headphones (54.92%), wireless in-ear headphones (53.41%) and Bluetooth speaker(s) (50.00%). However, wireless in-ear headphones (31.06%), and wired in-ear headphones (22.35%) were the leading monitoring devices most often used by listeners by a significant margin, followed by built-in smartphone speakers (13.64%), and then wireless over-ear headphones (12.88%).

**Table 5:** Monitoring devices participants have used (*N* = 264) and most often use (*N* = 264) to consume podcasts with a smartphone.

	**Monitoring devices used**			**Monitoring devices most often used**		
	**to consume podcasts**			**to consume podcasts**		
**Monitoring device**	**Frequency**	**%**	**As a % of sample**	**Frequency**	**%**	**As a % of device cases**
Built-in smartphone speakers	146	16.33	55.30	36	13.64	24.66
Wired in-ear headphones	145	16.22	54.92	59	22.35	40.69
Wireless in-ear headphones	141	15.77	53.41	82	31.06	58.16
Bluetooth speaker(s)	132	14.77	50.00	16	6.06	12.12
Built-in vehicle speakers	120	13.42	45.45	22	8.33	18.33
Wireless over-ear headphones	96	10.74	36.36	34	12.88	35.42
Wired over-ear headphones	92	10.29	34.85	9	3.41	9.78
Other speaker(s)^*^	9	1.01	3.41	2	0.76	22.22
Bone conduction headset	6	0.67	2.27	1	0.38	16.67
Other built-in device speakers^*^	4	0.45	1.52	1	0.38	25.00
Bluetooth hearing aids^*^	1	0.11	0.38	–	–	–
Other wireless headphones^*^	1	0.11	0.38	1	0.38	100.00
Other headphones^*^	1	0.11	0.38	1	0.38	100.00
Total	894	100.00	338.64	264	100.00	–
Monitoring device groups^**^						
Headphones devices	483	53.97	182.95	187	70.83	–
Loudspeakers devices	411	46.03	156.06	77	29.17	–

^*^Computed from *Other not listed (please specify)* responses. ^**^Computed from monitoring device responses according to analysis criteria outlined in the results section.

Additional analysis was conducted to explore how the monitoring devices participants used to listen were distributed between headphone- and loudspeaker-based devices. The *loudspeaker devices* group consisted of built-in smartphone speakers, Bluetooth speaker(s), built in vehicle speakers, other speaker(s), and other built-in device speakers devices. The *headphone devices* group comprised of all remaining devices listed in Table 5. This analysis found that 53.97% of all monitoring devices used by participants were headphones, while the remaining 46.03% were loudspeakers. Similarly, 70.83% of participants reported most often using headphones to listen, compared to 29.17% who most often used loudspeakers. 96.97% of participants reported having used at least one type of headphone monitoring device, while 18.94% of participants reported only having used headphones. In contrast, 81.06% of participants reported having used at least one loudspeaker monitoring device, while just 3.03% reported only having used loudspeakers to listen. 78.03% of participants had experience of using both headphones and loudspeakers.

#### 3.2.4. Multitasking activities

Multitasking activity response frequencies are shown in Table 6. The data presented in the “as a % of activity cases” column describes activities most often engaged in while consuming podcasts as a proportion of the total cases for each activity. The results found that 3.03% of participants didn't engage in multitasking, choosing instead to focus solely on the listening experience. There was also very little difference observed in listeners' multitasking habits between average weekdays and weekend days. *Doing housework* (76.89%), *preparing food* (73.86%) and *exercising* (61.74%) were the top three activities engaged in by the most participants. These were also the top three activities that participants reported most often engaging in, and while *doing housework* (26.89%) was still the most popular activity, the order of *exercising* (23.95%) and *preparing food* (10.92%) was reversed.

**Table 6:** Activities participants have engaged in (*N* = 264) and most often engage in (*N* = 238) while listening to podcasts with a smartphone.

	**Activities engaged in while**			**Activities most often engaged**		
	**consuming podcasts**			**in while consuming podcasts**		
**Multitasking activity**	**Frequency**	**%**	**As a % of sample**	**Frequency**	**%**	**As a % of activity cases**
Doing housework	203	18.45	76.89	64	26.89	31.53
Preparing food	195	17.73	73.86	26	10.92	13.33
Exercising	163	14.82	61.74	57	23.95	34.97
Messaging *via* phone/computer	116	10.55	43.94	5	2.10	4.31
Using social media sites	112	10.18	42.42	15	6.30	13.39
Other computer activities	83	7.55	31.44	15	6.30	18.07
Playing video games	39	3.55	14.77	6	2.52	15.38
Driving^*^	30	2.73	11.36	11	4.62	36.67
Walking^*^	27	2.45	10.23	11	4.62	40.74
Making Art/Crafting^*^	17	1.55	6.44	5	2.10	29.41
Shopping/Running Errands^*^	15	1.36	5.68	2	0.84	13.33
Working^*^	14	1.27	5.30	6	2.52	42.86
Showering/Bathing^*^	12	1.09	4.55	3	1.26	25.00
Reading	11	1.00	4.17	1	0.42	9.09
Commuting^*^	10	0.91	3.79	3	1.26	30.00
Going to sleep^*^	9	0.82	3.41	2	0.84	22.22
No activity^****^	8	0.73	3.03	–	–	–
Watching TV	5	0.45	1.89	2	0.84	40.00
Using public transport^*^	5	0.45	1.89	1	0.42	20.00
Gardening^*^	5	0.45	1.89	1	0.42	20.00
DIY^*^	4	0.36	1.52	–	–	–
Other Travel^*^	3	0.27	1.14	–	–	–
Cycling^*^	3	0.27	1.14	1	0.42	33.33
Eating^*^	3	0.27	1.14	–	–	–
Watching films	2	0.18	0.76	–	–	–
Doing puzzles^*^	2	0.18	0.76	–	–	–
Childcare^*^	2	0.18	0.76	1	0.42	50.00
Listening to music	1	0.09	0.38	–	–	–
Talking on the phone	1	0.09	0.38	–	–	–
Total	1,100	100.00	416.67	238	100.00	–
**Multitasking activity groups** ^**^						
Work activities	438	39.82	165.91	100	42.02	–
Media activities	370	33.64	140.15	44	18.49	–
Leisure activities	206	18.73	78.03	67	28.15	–
Transit activities	78	7.09	29.55	27	11.34	–
No activity	8	0.73	3.03	–	–	–
**Removed**						
Other not listed (invalid)^***^	–	–	–	18	–	–
No activity (invalid)^*****^	–	–	–	8	–	–

^*^Computed from *Other not listed (please specify)* responses. ^**^Computed from multitasking activity responses according to analysis criteria outlined in the results section. ^***^Removed as multiple/void free text responses were provided for activity most often engaged in. ^****^Participant reported not engaging in any multitasking activities. ^*****^Removed due to participants not engaging with any multitasking activity.

Additional analysis was also conducted to further explore how participants engaged in different multitasking activity modalities. Activity responses were separated into four categories representing *work, leisure, media*, and *transit activities*. The *work activities* group consisted of the *doing housework, preparing food, working, shopping/running errands, gardening, DIY*, and *childcare* responses. The *media activities* group included *watching TV, reading, listening to music, walking on the phone, sending messages via phone or computer, using social media sites, watching films, other computer activities*, and *playing video games*. The *leisure activities* group consisted of *exercising, making art/crafting, showering/bathing, going to sleep, eating*, and *doing puzzles*. The *transit activities* group consisted of *driving, using public transport, walking, other travel, commuting*, and *cycling*.

This analysis found that most activities were *work* related, accounting for 39.82% of all activities engaged in and 42.02% of activities that participants most often engaged in. This was followed by the *media* modality which accounted for 33.64% of all activities engaged in, but only 18.49% of the activities most often engaged in. This was contrasted by the *leisure* related activities modality, which, despite representing just 18.73% of all activities engaged in, accounted for 28.15% of activities most often engaged in. Lastly, *transit* related activities received by far the lowest proportion of participant engagement, accounting for only 7.09% of all activities and 11.34% of activities most often engaged in.

The proportion of time participants spent multitasking while listening on average weekday and weekend days is also shown in Table 7. These results revealed that the majority of participants engage in multitasking activities for at least 90% of the time they spend listening to podcasts, while only 3.79 and 8.33% reported never engaging in multitasking activities on weekdays and weekend days, respectively.

**Table 7:** Proportion of time participants spend multitasking while listening to podcasts using a smartphone on average weekdays and weekend days (*N* = 264).

	**Average weekday**		**Average weekend day**	
**Multitasking proportion %**	**Frequency**	**%**	**Frequency**	**%**
100	116	43.94	119	45.08
90	41	15.53	32	12.12
80	31	11.74	20	7.58
70	20	7.58	19	7.20
60	4	1.52	5	1.89
50	22	8.33	22	8.33
40	4	1.52	4	1.52
30	8	3.03	9	3.41
20	5	1.89	6	2.27
10	3	1.14	6	2.27
0	10	3.79	22	8.33
Total	264	100.00	264	100.00

#### 3.2.5. Podcast discovery methods

Podcast discovery method response frequencies are shown in Table 8. The data presented in the “as a % of method cases” column describes methods most often used to discover podcasts as a proportion of the total cases for each method. The results showed that consumers use a wide variety of methods to discover podcasts. The majority of the sample had experience of using *recommendations from friends & family* (71.29%), *listening to podcasts* (68.94%), and *streaming services* (59.09%), while a significant proportion also had experience of *searching the internet* (48.86%), and using *recommendations on social media* (47.73%). Similarly, there was a fairly even split between *recommendations from friends & family* (23.66%), *streaming services* (23.66%), and *listening to podcasts* (19.85%) for the three methods most often used by participants.

**Table 8:** Methods participants have used (*N* = 264) and most often use (*N* = 262) to discover podcasts.

	**Methods used to**			**Methods most often used to**		
	**discover podcasts**			**to discover podcasts**		
**Discovery method**	**Frequency**	**%**	**As a % of sample**	**Frequency**	**%**	**As a % of method cases**
Recommendations from friends/family	189	20.09	71.59	62	23.66	32.80
Listening to podcasts	182	19.34	68.94	52	19.85	28.57
Streaming services	156	16.58	59.09	62	23.66	39.74
Searching the internet	129	13.71	48.86	28	10.69	21.71
Recommendations on social media	126	13.39	47.73	20	7.63	15.87
Listening to radio	57	6.06	21.59	–	–	–
Recommendations from YouTube creators	38	4.04	14.39	6	2.29	15.79
Recommendations from YouTube watch history	19	2.02	7.20	4	1.53	21.05
Podcast player app^*^	13	1.38	4.92	5	1.91	38.46
Newsletters^*^	9	0.96	3.41	5	1.91	55.56
Recommendations from industry media^*^	7	0.74	2.65	–	–	–
Recommendations from print media^*^	6	0.64	2.27	2	0.76	33.33
Recommendations from online media^*^	3	0.32	1.14	1	0.38	33.33
Recommendations from podcasters^*^	3	0.32	1.14	1	0.38	33.33
Recommendations from colleagues^*^	2	0.21	0.76	–	–	–
Recommendations from television^*^	1	0.11	0.38	–	–	–
Podcasting awards^*^	1	0.11	0.38	–	–	–
Total	941	100.00	356.44	262	100.00	–
Podcast discovery method groups^**^						
Other online media	332	35.28	125.76	66	25.19	–
Personal recommendations	191	20.30	70.35	62	23.66	–
Podcasts	185	19.66	70.08	53	20.23	–
Podcast apps	169	17.96	64.02	67	25.57	–
Offline media	64	6.80	24.24	14	5.34	–
**Removed**						
Other not listed (invalid)^***^	–	–	–	2	–	–

^*^Computed from *Other not listed (please specify)* responses. ^**^Computed according to analysis criteria outlined in the results section. ^***^Removed as multiple/void free text responses were provided for discovery method most often used.

Additional analysis was also conducted to provide a deeper insight into how listeners utilize different methods of consumption in podcast discovery. Method responses were separated into five categories based on research conducted by Insights (2019), including *other online media, personal recommendations, podcasts, podcast apps*, and *offline media*. The *other online media* group consisted of responses to *searching the internet, recommendations from social media, YouTube creators, YouTube viewing history, online media, podcasting awards, newsletters*, and *industry media*. The *personal recommendations* group included the *recommendations from friends & family* and *colleagues responses*. The *podcasts* group comprised responses from the *listening to podcasts* and *recommendations from podcast hosts* items. The *podcast apps* group included responses from the *streaming services* and *podcast apps* items. The *offline media* group consisted of the *listening to radio, recommendations from print media*, and *recommendations from television* items.

The results from this group analysis showed that while the *other online media* group represented the clear majority share of all methods used (35.28%), the methods participants most often used were distributed relatively equally between *podcast apps* (25.57%), *other online media* (25.19%), *personal recommendations* (23.66%), and *podcasts* (20.23%). *Offline media* related methods were by far the least common amongst the sample, with only 6.8% of participants having used an *offline media* method, and 5.34% providing one as the method they had most often used.

#### 3.2.6. Correlations amongst aspects of podcast listening engagement

A series of Spearman's rank-order and Pearson's product-moment correlations were run to assess all pairwise relationships amongst the *aspects of podcast listening engagement*. As the two survey questions concerning the average amount of time participants spent listening to podcasts on weekday and weekend days were measured using a non-continuous ordinal scale and the other *aspects of podcast listening engagement* metrics were continuous data, a series of Spearman's rank-order correlations were run to assess the relationships between these variables (Schober et al., 2018). These correlations are shown in Table 9. Preliminary analysis, consisting of visual inspections of scatterplots, found all pairwise relationships between the amount of listening time variables and the other *aspects of podcast listening engagement* to be monotonic, with the exception of both amount of listening time variables and the total monitoring devices used pairings.

**Table 9:** Spearman's rank-order correlations amongst the aspects of podcast listening engagement (*N* = 264).

		**Weekday**	**Weekend**	**Total**	**Total**	**Total**	**Total**
		**listening**	**listening**	**locations**	**devices**	**activities**	**discovery**
Weekday	Rho	–					
listening	Sig.	–					
Weekend	Rho	0.551^*^	–				
listening	Sig.	<0.001	–				
Total	Rho	0.303^*^	0.387^*^	–			
locations	Sig.	<0.001	<0.001	–			
Total	Rho	0.006	0.161^*^	0.415^*^	–		
devices	Sig.	0.926	0.009	<0.001	–		
Total	Rho	0.235^*^	0.296^*^	0.494^*^	0.340^*^	–	
activities	Sig.	<0.001	<0.001	<0.001	<0.001	–	
Total	Rho	0.198^*^	0.176^*^	0.306^*^	0.322^*^	0.309^*^	–
discovery	Sig.	0.001	0.004	<0.001	<0.001	<0.001	–

^*^Correlation is significant at the 0.01 level (2-tailed).

There was a statistically significant, moderate positive correlation between average amount of weekday listening time and the total number of locations in which podcasts were listened to, *r*_(262)_ = 0.30, *p* < 0.001. There were also two statistically significant, small positive correlations between weekday listening time and the total number of activities simultaneously engaged in while listening, *r*_(262)_ = 0.24, *p* < 0.001, and the number of methods used to discover podcasts, *r*_(262)_ = 0.20, *p* < 0.001, respectively. A statistically significant, moderate positive correlation was also found between the average amount of weekend day listening time and total listening locations, *r*_(262)_ = 0.39, *p* < 0.001. Similarly, there were also two statistically significant, small positive correlations between weekend day listening time and the total number of activities simultaneously engaged in, *r*_(262)_ = 0.30, *p* < 0.001, and the number of methods used to discover podcasts, *r*_(262)_ = 0.18, *p* < 0.004, respectively.

As the computed total listening locations, total monitoring devices, total multitasking activities, and total discovery methods per participant metrics were continuous data, a series of Pearson's product-moment correlations were run to assess all pairwise relationships between these variables (Schober et al., 2018). These correlations are shown in Table 10. Preliminary analysis found all pairwise relationships to be linear with all variables normally distributed, as assessed by visual inspection of Normal Q–Q Plots. Additionally, assessment of scatterplots for the bivariate combinations found there were no outliers.

**Table 10:** Pearson's product-moment correlations amongst the total monitoring devices, total multitasking activities, total listening locations, and total discovery methods. (*N* = 264).

		**Total**	**Total**	**Total**	**Total**
		**devices**	**activities**	**locations**	**discovery**
Total	PCC	–			
devices	Sig.	–			
Total	PCC	0.352^*^	–		
activities	Sig.	<0.001	–		
Total	PCC	0.446^*^	0.498^*^	–	
locations	Sig.	<0.001	<0.001	–	
Total	PCC	0.303^*^	0.318^*^	0.308^*^	–
discovery	Sig.	<0.001	<0.001	<0.001	–

^*^Correlation is significant at the 0.01 level (2-tailed).

There were three statistically significant, moderate positive correlations between the total number of locations in which podcasts were listened to and the number of activities simultaneously engaged in while listening, *r*_(262)_ = 0.50, *p* < 0.001, the number of monitoring devices used to listen, *r*_(262)_ = 0.45, *p* < 0.001, and the number of methods used to discover podcasts, *r*_(262)_ = 0.31, *p* < 0.001, respectively. There were also two statistically significant, moderate positive correlations between the total number of activities simultaneously engaged in while listening to podcasts and the total number of monitoring devices used to listen, *r*_(262)_ = 0.35, *p* < 0.001, and the number of methods used to discover podcasts, *r*_(262)_ = 0.32, *p* < 0.001, respectively. Finally, there was a statistically significant, moderate positive correlation between the total number of methods used to discover podcasts and the number of monitoring devices used to listen, *r*_(262)_ = 0.30, *p* < 0.001.

Collectively, there were a total of twelve statistically significant relationships between the different aspects of measured podcast listening engagement, consisting of two moderate positive and four small positive Spearman's rank-order correlations, and six moderate positive Pearson's product moment correlations. Therefore, as there were several statistically significant positive correlations observed amongst the *aspects of podcast listening engagement* the null hypothesis can be rejected and the alternate hypothesis [H2] accepted.

### 3.3. Relationships between environmental context factor scores and aspects of podcast listening engagement

To answer the third research question, which asked how the proposed *factors of environmental context* relate to the *aspects of podcast listening engagement*, a series of Spearman's rank-order and Pearson's product-moment correlations were run to assess the pairwise relationships between the *environmental context factor scores* and *aspects of podcast listening engagement*.

As the two questions that asked participants to report the average amount of time they spent listening to podcasts on weekday and weekend days were measured using a non-continuous ordinal scale and the *environmental context factor scores* were continuous data, a series of Spearman's rank-order correlations were run to assess the relationships between these variables (Schober et al., 2018). These correlations are shown in Table 11. Preliminary analysis, consisting of visual inspections of scatterplots, found all pairwise relationships between the amount of listening time variables and factor scores to be monotonic, with the exception of the weekend listening and *exercise* factor score pairing.

**Table 11:** Spearman's rank-order correlations amongst the aspects of podcast listening engagement & environmental context factor scores (*N* = 264).

		**Weekday**	**Weekend**	**Total**	**Total**	**Total**	**Total**	**Outdoors**	**Indoors &**	**Evenings**	**Soundscape**	**Exercise**	**Overall**
		**listening**	**listening**	**devices**	**activities**	**locations**	**discovery**	**FS**	**at home FS**	**FS**	**& at work FS**	**FS**	**mean FS**
Weekday	Rho	–											
listening	Sig.	–											
Weekend	Rho	0.551^**^	–										
listening	Sig.	<0.001	–										
Total	Rho	0.006	0.161^**^	–									
devices	Sig.	0.926	0.009	–									
Total	Rho	0.235^**^	0.296^**^	0.340^**^	–								
activities	Sig.	<0.001	<0.001	<0.001	–								
Total	Rho	0.303^**^	0.387^**^	0.415^**^	0.494^**^	–							
locations	Sig.	<0.001	<0.001	<0.001	<0.001	–							
Total	Rho	0.198^**^	0.176^**^	0.322^**^	0.309^**^	0.306^**^	–						
discovery	Sig.	0.001	0.004	<0.001	<0.001	<0.001	–						
Outdoors	Rho	0.054	0.097	−0.07	0.008	0.128^*^	0.021	–					
FS	Sig.	0.386^***^	0.116^***^	0.258^***^	0.901^***^	0.038^***^	0.73^***^	–					
Indoors &	Rho	0.074	0.126^*^	0.072	0.153^*^	0.096	0.073	0.509^**^	–				
at home FS	Sig.	0.231^***^	0.041^***^	0.243^***^	0.013^***^	0.122^***^	0.239^***^	<0.001^***^	–				
Evenings	Rho	0.127^*^	0.250^**^	0.012	0.067	0.126^*^	0.107	0.252^**^	0.317^**^	–			
FS	Sig.	0.039^***^	<0.001^***^	0.846^***^	0.28^***^	0.04^***^	0.082^***^	<0.001^***^	<0.001^***^	.			
Soundscape	Rho	0.240^**^	0.305^**^	0.097	0.205^**^	0.295^**^	0.046	0.293^**^	0.236^**^	0.234^**^	–		
& at work FS	Sig.	<0.001^***^	<0.001^***^	0.115^***^	<0.001^***^	0.001^***^	0.454^***^	<0.001^***^	<0.001^***^	<0.001^***^	–		
Exercise	Rho	0.089	0.132^*^	0.018	0.081	0.219^**^	0.009	0.263^**^	0.233^**^	0.186^**^	0.335^**^	–	
FS	Sig.	0.148^***^	0.032^***^	0.774^***^	0.188^***^	<0.001^***^	0.889^***^	<0.001^***^	<0.001^***^	0.002^***^	<0.001^***^	–	
Overall	Rho	0.126^*^	0.266^**^	0.055	0.160^**^	0.262^**^	0.102	0.699^**^	0.661^**^	0.581^**^	0.622^**^	0.589^**^	–
mean FS	Sig.	0.041^***^	<0.001^***^	0.371^***^	0.009^***^	<0.001^***^	0.098^***^	<0.001^***^	<0.001^***^	<0.001^***^	<0.001^***^	<0.001^***^	–

^*^Correlation is significant at the 0.05 level (2-tailed). ^**^Correlation is significant at the 0.01 level (2-tailed). ^***^FS = Factor Scores.

There were two statistically significant, small positive correlations between the average amount of weekday listening time, and the *soundscape & at work* factor scores, *r*_(262)_ = 0.24, *p* < 0.001, and the computed *overall mean* factor scores, *r*_(262)_ = 0.13, *p* < 0.041, respectively. A statistically significant, moderate positive correlation was found between the average amount of weekend day listening time and the *soundscape & at work* factor scores, *r*_(262)_ = 0.31, *p* < 0.001. While there were four statistically significant, small positive correlations between weekend day listening time and *overall mean*, *r*_(262)_ = 0.27, *p* < 0.001, *evenings*, *r*_(262)_ = 0.25, *p* < 0.001, *exercise*, *r*_(262)_ = 0.13, *p* < 0.032, and *indoors & at home* factor scores, *r*_(262)_ = 0.13, *p* < 0.041, respectively.

As the *environmental context factor scores* and the computed total listening locations, total monitoring devices, total multitasking activities, and total discovery methods per participant metrics were all continuous data, a series of Pearson's product-moment correlations were run to assess all pairwise relationships between these variables (Schober et al., 2018). These correlations are shown in Table 12. Preliminary analysis found all pairwise relationships were linear with all variables normally distributed, as assessed by visual inspection of Normal Q–Q Plots. Assessment of bivariate scatterplots found there were no outliers.

**Table 12:** Pearson's product-moment correlations amongst the total monitoring devices, total multitasking activities, total listening locations, total discovery methods, and environmental context factor scores (*N* = 264).

		**Total**	**Total**	**Total**	**Total**	**Outdoors**	**Indoors &**	**Evenings**	**Soundscape**	**Exercise**	**Overall**
		**devices**	**activities**	**locations**	**discovery**	**FS**	**at home FS**	**FS**	**& at work FS**	**FS**	**mean FS**
Total	PCC	–									
devices	Sig.	–									
Total	PCC	0.352^**^	–								
activities	Sig.	<0.001	–								
Total	PCC	0.446^**^	0.498^**^	–							
locations	Sig.	<0.001	<0.001	–							
Total	PCC	0.303^**^	0.318^**^	0.308^**^	–						
discovery	Sig.	<0.001	<0.001	<0.001	–						
Outdoors	PCC	−0.074	−0.02	0.115	0.029	–					
FS	Sig.	0.23^***^	0.751^***^	0.062^***^	0.643^***^	–					
Indoors &	PCC	0.033	0.138^*^	0.107	0.081	0.457^**^	–				
at home FS	Sig.	0.596^***^	0.025^***^	0.084^***^	0.188^***^	<0.001^***^	–				
Evenings	PCC	0.01	0.056	0.115	0.115	0.207^**^	0.263^**^	–			
FS	Sig.	0.874^***^	0.366^***^	0.061^***^	0.062^***^	<0.001^***^	<0.001^***^	–			
Soundscape	PCC	0.092	0.186^**^	0.284^**^	0.052	0.313^**^	0.258^**^	0.238^**^	–		
& at work FS	Sig.	0.138^***^	0.002^***^	<0.001^***^	0.397^***^	<0.001^***^	<0.001^***^	<0.001^***^	–		
Exercise	PCC	0.014	0.028	0.216^**^	0.027	0.244^**^	0.205^**^	0.146^*^	0.334^**^	–	
FS	Sig.	0.822^***^	0.655^***^	<0.001^***^	0.666^***^	<0.001^***^	<0.001^***^	0.018^***^	<0.001^***^	–	
Overall	PCC	0.023	0.121	0.261^**^	0.095	0.691^**^	0.679^**^	0.577^**^	0.667^**^	0.600^**^	–
mean FS	Sig.	0.709^***^	0.05^***^	<0.001^***^	0.125^***^	<0.001^***^	<0.001^***^	<0.001^***^	<0.001^***^	<0.001^***^	–

^*^Correlation is significant at the 0.05 level (2-tailed). ^**^Correlation is significant at the 0.01 level (2-tailed). ^***^FS = Factor Scores.

There were three statistically significant, small positive correlations between the total locations in which podcasts were listened to, and the *soundscape masking & at work*, *r*_(262)_ = 0.28, *p* < 0.001, computed *r*_(262)_ = 0.26, *p* < 0.001, and *exercise* factor scores, *r*_(262)_ = 0.22, *p* < 0.001, respectively. There were also two statistically significant, small positive correlations between the total number of multitasking activities engaged in and the *soundscape masking & at work*, *r*_(262)_ = 0.19, *p* < 0.002, and *indoors & at home* factor scores, *r*_(262)_ = 0.14, *p* < 0.025, respectively.

In summary, there was a total of twelve statistically significant relationships between the measured *aspects of podcast listening engagement* and *environmental context factor scores*, consisting of one moderate positive and six small positive Spearman's rank-order correlations, and five small positive Pearson's product moment correlations. Consequently, as there were several statistically significant positive correlations amongst the *aspects of podcast listening engagement* and the proposed *factors of environmental context* the null hypothesis can be rejected and the alternate hypothesis [H3] accepted.

## 4. Discussion

This section comprises of a discussion of the results structured first around the three tested hypotheses [H1], [H2], and [H3], followed by an evaluation of the potential implications of the work for the fields of podcast studies, media personalisation, and attentional processing research.

### 4.1. Hypothesis 1: Factors of environmental context

#### 4.1.1. Factor interpretations

The EFA conducted in the present study permitted the acceptance of hypothesis [H1] by uncovering the presence of five latent variables representing factors of environmental context that influence attentional engagement in podcast listening experiences. These are shown together with Likert response frequencies in Figure 1. Factors are organized in order of most variance explained and items in order of the strongest loadings within each factor. The following section combines results from the factor loadings presented in Table 1 with Likert response results shown in Figure 1, free text response data from participants collected in the qualitative section of the study, and findings from existing literature, to produce interpretations for the five factors produced by the analysis.

Analysis of the *environmental context factor scores* and factor correlations shown in Table 2 indicate that there is considerable variation in participants' scores between the same factors. As such, the interpretations detailed in this analysis are presented with the understanding that the factors they represent influence different sections of the sampled population in different ways.

It is also noted that the results from this EFA should be subject to independent validation *via* further confirmatory factor analysis studies. Irrespective of this it is also important to consider that the results should not be considered a comprehensive model of environmental context factors that influence attentional engagement. The *factors of environmental context* produced by the EFA were derived from an initial 30-item scale originally presented to participants in the form of an online survey, and as such the findings from this analysis do not preclude additional factors from being identified and proposed by further analysis in future studies.

#### 4.1.2. Outdoors

The first factor was labeled *Outdoors* and explained 12.84% of the total variance amongst all items. All four of the outdoor listening items included in the initial scale loaded together onto the factor, indicating that differences in how outdoor atmospheric conditions were perceived as influencing attentional engagement were not significant enough to cause any of the items not to load. The *outdoors in light, cold*, and *warm conditions* items all exhibited similarly strong loadings and positive Likert ratings with *light* conditions loading most strongly, whereas the *outdoors in dark conditions* item registered a comparatively weaker loading and less positive Likert scale ratings. This was reflected by some of the free text responses provided by participants who reported experiencing lower attentional engagement when listening in outdoor public spaces in dark conditions, with the primary reason being a concern for their personal safety.

This interpretation is consistent with previous results (McGill et al., 2020) that investigated the influence of acoustic transparency on auditory mixed reality soundscapes, finding that nearly half of participants believed their safety was compromised when wearing noise canceling headphones that occluded environmental noise, as opposed to acoustically transparent headphones that enabled enhanced perception of external stimuli in the surrounding soundscape while listening.

The *cold* and *warm conditions* item factor loadings and Likert response data suggested that temperature had minimal influence over attentional engagement. However, research suggests that while the optimum environmental temperature for higher attention changes only slightly over time from cooler to warmer, both extreme hot and cold temperatures are found to significantly lower focused attentional ability (Choi et al., 2019). As such further research is required to fully explore the influence of environmental temperature over attentional engagement in the podcast listening experience.

#### 4.1.3. Indoors & at home

The second factor was labeled *Indoors & at home* and explained 12.17% of the total variance across all items. All four of the indoors items included in the initial scale loaded together onto the factor, again indicating that differences in how indoor atmospheric conditions were perceived as influencing attentional engagement were not significant enough to cause any of the items not to load. The *at home* item also loaded on to this factor, indicating that the factor was more specifically associated with listening at home as opposed to indoor environments more generally.

The *indoors in warm conditions* and *light conditions* exhibited by far the strongest loadings. The *at home* item was the next strongest loading with the *warm conditions, light conditions*, and *at home* items all receiving similarly highly positive Likert scale ratings. The fourth strongest item loading, *indoors in cold conditions*, received the least positive Likert ratings in the factor, suggesting that cold conditions had more of a negative influence on attentional engagement when listening indoors rather than outdoors.

Conversely, the *indoors in dark conditions* item was the weakest loading on the factor, despite receiving similarly positive Likert ratings to the *indoors in warm, light*, and *cold conditions* items. The *indoors in dark conditions* item Likert ratings were noticeably more positive compared to the *outdoors in dark conditions* item, further supporting the interpretation of the first factor that some participants experience lower attentional engagement when listening outdoors in dark conditions due to concerns over their personal safety.

Collectively, the *Indoors & at home* items received the most positive Likert ratings of all the factors, suggesting that the *Indoors & at home* factor is the most closely associated with higher attentional engagement. The results from the listening location questions in the present study, and a digital media consumer survey (Edison Research, 2019), both indicated that the most common location in which podcasts were consumed was the home. When considered alongside the findings of Chan-Olmsted and Wang (2020), that characterized podcast listening at home as more active and instrumental (Rubin, 1984), these results might suggest that higher attentional engagement is more closely associated with instrumental consumption where the focus of interest is centered around the specific content, than habitual ritualized consumption where the focus is centered on the medium (Rubin and Perse, 1987).

#### 4.1.4. Evenings

The third factor was labeled *Evenings* and explained 11.30% of the total variance across all items. All four of the *evenings* items included in the initial scale loaded together onto the factor, with all of the remaining time related *morning* and *daytime* items failing to load.

The *weekend days in the late evening* and *weekdays in the late evening* items represented the strongest loadings on the factor respectively, yet notably received less positive attentional engagement Likert ratings compared to the *weekend days in the early evening* and *weekdays in the early evening* items. Despite receiving the most positive Likert ratings within the factor the *weekdays in the early evening* item registered by far the weakest loading on the factor. Furthermore, an inverse relationship was observed between the strength of loading and Likert ratings for each item, prompting the observation that items most strongly associated with the *evenings* factor were less positively associated with being actively engaged.

These results could potentially be explained, in part, by the free text qualitative responses provided by 12 participants who reported listening to podcasts when trying to fall asleep. Findings from the infinite dial podcast consumer report (Edison Research, 2019) would also support this interpretation, with statistics showing that 51% of participants surveyed had listened to podcasts when relaxing before going to sleep. This was also mirrored by open ended responses provided to a study (Best and Cole, 2022) exploring young people's engagement with podcasts that indicated some young people use podcasts to help them relax and fall asleep. In fact, the popularity of the use of podcasts as a sleep aid has grown to such an extent that it is now being reflected back by creators and industry writers with podcasts being specifically produced and marketed to satiate the audience's desire for sleep inducing sounds (Hunt, 2021).

It is also possible that the natural circadian rhythms that serve to regulate various physical, mental and behavioral changes in an individual over a 24 h cycle could influence their capacity for higher attentional engagement at different times of the day. Valdez (2019) conducted a study investigating the influence of circadian rhythms on the four components of attention outlined in the model proposed by Posner and Rafal (1987). Circadian rhythms were observed in all four components: tonic alertness, phasic alertness, selective attention, and sustained attention. Overall attention was found to increase throughout the day and was at it's lowest at night and during the early morning. A review of current work relating to the influence of circadian rhythms on different aspects of auditory research concluded that circadian aspects should be given greater consideration when designing auditory experiments due to the growing breadth of experimental evidence linking circadian variations to the central and peripheral auditory systems (Cederroth et al., 2020).

With the exception of the *weekend days in the morning item*, all of the remaining time related items that failed to load on the factor received similarly positive Likert ratings compared to the evening items that did load. This suggests that while most participants reported experiencing largely positive attentional engagement for each time period on an individual basis, collectively it appears that the evening items were the only time related loadings with enough shared covariance to be considered an underlying factor.

#### 4.1.5. Soundscape & at work

The fourth factor was labeled *Soundscape & at work* and explained 9.35% of the total variance amongst all items. The *high levels of background noise* item was the strongest loading, followed by the *moderate levels of background noise, environments mainly comprised of mechanical sounds, environments mainly comprised of sounds that indicate the presence of humans*, and the *at work* items. The *low levels of background noise* and *environments mainly comprised of natural soundscape* items did not load. Four of the five items that loaded on the factor, the *high background noise, mechanical soundscape, human soundscape*, and *at work* items, received by far the most negative individual Likert ratings across all of the 30 items in the initial scale. However, the *low background* noise item received the single most positive Likert rating and the *natural soundscape* item was also reviewed favorably, despite both not loading on the factor. These results suggest that listeners tend to experience lower attentional engagement when consuming podcasts in soundscapes containing a higher concentration of disturbing stimuli. This is consistent with the results of a study by Smith (1991) that investigated how noise affected participants' performance in focused attention and cognitive search tasks, finding that intrusive noise impeded performance in a focused attention task.

However, it is notable that a significant proportion of responses to items that represented disturbing stimuli were positively associated with higher attentional engagement. Therefore, it is argued that the *soundscape & at work* factor could also be indicative of some listeners purposefully using podcasts, together with the occlusion provided by headphones, to mask disturbing elements of their surrounding soundscape and increase their capacity to experience higher attentional engagement within their own personal listening bubble.

This interpretation is consistent with research conducted by Herrmann and Johnsrude (2020) which found that, over time, some listeners experience increased levels of absorption and enjoyment when listening to stories masked by multitalker babble. The interpretation could also potentially be explained further by a participant's response to the first qualitative question, who reported listening to familiar voices on their favorite podcasts to make them feel safer when they felt overwhelmed or lonely in public spaces. This response is indicative of a form of parasocial engagement where listeners form deep social bonds with hosts of their favorite podcast shows (Schlütz and Hedder, 2021) and personas in the wider broadcast media more generally (Vickery and Ventrano, 2020). The formation of this factor is also consistent with analysis of the monitoring device results, shown in Table 5, that found the majority of participants (70.83%) most often used a headphone-based device to consume podcasts.

The results from the present study highlight the need for future work exploring how the acoustic transparency and occlusion of different headphone-based monitoring devices mediates listeners' attentional engagement in mobile listening experiences. Such research would be especially relevant to future studies investigating the soundscape masking as a function of podcast listening engagement interpretation proposed for this factor.

#### 4.1.6. Exercise

The fifth factor was labeled *Exercise* and explained 9.02% of the total variance across all items. The *on a run* item was the strongest loading of any in the EFA and received similarly positive Likert ratings to the *at the gym* and *on a walk in a rural environment* items, both registering successively weaker loadings than the last. It was notable that the other item representing a form of exercise in the initial scale, *on a walk in an urban environment*, failed to load on this factor. This suggests that the factor was more closely associated with forms of exercise engaged in for the purposes of pleasure and wellbeing as opposed to more functional affordances of exercise generally associated with urban environments such as commuting or running errands. This would also support the assertion that the attentional engagement manifested in this factor is associated with the restorative influence of natural environments and stimuli on attentional engagement and cognitive control (Berman et al., 2008).

The *exercise* factor may also be indicative of the findings of research conducted by Pontifex et al. (2015) that investigated the influence of exercise on attentional processes. The study found that a single bout of aerobic physical exercise had the effect of sustaining attentional processing, relative to a prolonged period of sedentary inactivity. It could be hypothesized that a relationship may exist between attentional processing ability and attentional engagement in podcast listening experiences.

### 4.2. Hypothesis 2: Associations amongst aspects of podcast listening engagement

The results of correlation tests that were run to investigate the associations amongst the *aspects of podcast listening engagement*, and permit the testing of Hypothesis [H2], are shown in Tables 9, 10. They suggest that individuals who listen for longer periods of time on weekdays and weekend days tend to consume podcasts in a greater number of locations, engage in a larger number of multitasking activities while listening, and use a greater number of methods to discover podcasts, with weekend day listening more strongly correlated with the number of listening locations and multitasking activities compared to weekday listening. The results also suggest that individuals who listen in a larger number of locations tend to engage in more multitasking activities, use a greater number of monitoring devices, and a larger number of discovery methods. The results would also support the assertion that individuals who engage in a greater number of multitasking activities tend to use a larger number of monitoring devices and discovery methods. Finally, the results also suggest that listeners who use a greater number of methods to discover podcasts also tend to use a larger number of devices to listen.

These results support the acceptance of hypothesis [H2], showing that there are positive correlations between most of the *aspects of podcast listening engagement* measured in the present study. They build on the several aspects of podcast listening originally proposed by Tobin and Guadagno (2022) to form five additional *aspects of podcast listening engagement* that constitute a congruous suite of engagement metrics that rise and fall together to represent higher or lower levels of listener engagement.

### 4.3. Hypothesis 3: Associations amongst environmental context factor scores and aspects of podcast listening engagement

The results of correlation tests that were run to investigate the associations amongst the *environmental context factor scores* and *aspects of podcast listening engagement* are shown in Tables 11, 12. They suggest that listeners who experience higher *overall* attentional engagement tend to listen to podcasts for longer periods of time on both weekday and weekend days and also listen in a greater number of locations. Within this, the results for the constituent factors suggest that listeners who experience higher *soundscape & at work* factor attentional engagement tend to listen for longer periods of time on weekday and weekend days, listen in a greater number of locations and engage in a larger number of activities while listening, with weekend day listening more strongly correlated with *soundscape & at work* factor attentional engagement compared to weekday listening. The results also suggest that individuals who experience higher *exercise* factor attentional engagement tend to listen for longer periods of time on both weekday and weekend days. Finally, the results suggest that listeners who experience higher *evenings* factor attentional engagement tend to listen for longer periods of time on weekend days only.

These results support the acceptance of hypothesis [H3], showing that that there are positive correlations between many of the *aspects of podcast listening engagement* and *environmental context factor scores* surveyed in the present study. The *soundscape & at work* factor was found to be most closely associated with the *aspects of podcast listening engagement*, suggesting that the environmental soundscape that surrounds a listener is an especially important mediating factor for multiple facets of their podcast listening engagement. The results strongly support the assertion that consumers who listen for longer tend to experience higher attentional engagement. The finding that consumers who engage in a greater number of unique multitasking activities while listening tend to experience higher *soundscape & at work* factor attentional engagement is consistent with the *soundscape & at work* factor interpretation, in describing a factor of environmental context born of the listener's desire to audibly mask and move their attention away from disturbing sounds from stimuli in their surrounding environment.

Similarly, the finding that consumers who listen in a larger number of locations tend to experience higher *overall* and *soundscape & at work* factor attentional engagement further supports the *soundscape & at work* factor interpretation. Consumers who listen in a wider variety of locations are more likely to be exposed to potentially disturbing sounds in their surroundings and are therefore more likely to consume podcasts in their own personal listening bubble as a means of auditory masking and escape.

### 4.4. Implications for related research fields

#### 4.4.1. Podcast studies

The *aspects of podcast listening engagement* metrics were conceptualized by the present study for the primary purpose of investigating how the proposed *factors of environmental context* might be associated with a series of quantitative measures describing different facets of podcast listening engagement. However, the *aspects of podcast listening engagement* results could be highly pertinent to research in the field of podcast studies in their own right, providing new insights in podcast listening engagement, habits, and behaviors.

Greasley and Lamont (2006) found that highly engaged listeners were more likely to listen to a greater amount of music in everyday life listening experiences. When considered alongside this finding, analysis of the amount of listening time data in Table 3 might suggest that, as listeners tend to listen for shorter amounts of time on weekend days, they are less likely to experience higher attentional engagement on weekend days compared to weekdays. However, further analysis of the correlations between the *environmental context factor scores* and amount of listening time, shown in Table 11, found that every factor of environmental context exhibited a stronger positive association with weekend listening compared to weekday listening.

This suggests that while listeners are less likely to engage in longer episodes of podcast listening at the weekend, they tend to experience higher attentional engagement compared to weekday listening experiences. This could potentially be explained by differences in the average length of uninterrupted listening experiences between weekday and weekend listening, as opposed to the overall cumulative amount across a single day. However, as this information was not captured in the present survey, further research is required to test the validity of this hypothesis. This is a distinction highlighted by the results of a study conducted by Herrmann and Johnsrude (2020) that found listeners' absorption increased when repeatedly listening to multiple acoustically masked stories sequentially over a longer period of time.

Analysis of the listening location results in Table 4 suggests that while listeners possess a keen appetite for consuming podcasts in a wide variety of public locations, most consumers prefer to listen in private spaces that typically contain fewer disturbing environmental stimuli capable of negatively capturing their bottom-up attention and reducing attentional engagement. Further analysis indicates that while the vast majority of individuals consume podcasts in both public and private locations (87.88%), a small proportion of listeners only listen in either private (8.33%) or public (3.79%). Aside from reasons of personal preference, it's possible that listeners who only consume podcasts in private locations could be doing so due to personal circumstances that make it difficult for them to leave their home, or alternatively they might lack access to a headphone-based monitoring device that would allow them to listen in public spaces without disturbing others. In contrast, those who only listen in public spaces might do so as part of a daily routine while commuting or running errands. Further research is required to fully understand the listening behaviors observed in this data.

Analysis of the monitoring device results in Table 5 indicates that while participants tend to use a fairly equal number of different headphone- and loudspeaker-based devices to listen, the majority of participants most often consume podcasts using a headphone-based device. This finding is consistent with the assertions of Bull (2010) that podcast consumers like to create personal listening bubbles to partially separate themselves from stimuli in their environmental context. The results also support assertions made by Berry (2016), Heshmat et al. (2018), and Schlütz and Hedder (2021) that podcast listening is distinct from other audio-based media, as its strong associations with headphone listening contribute to a greater sense of intimacy in the listening experience.

The multitasking proportion results in Table 7 support the findings of Perks et al. (2019) that podcasting is a medium closely associated with multitasking, and is both consumed as a secondary activity that competes for the listener's attention alongside an array of other often multi-sensory activities, and also on occasion the sole activity occupying listeners' full attention (Chan-Olmsted and Wang, 2020). Analysis of the multitasking activity results shown in Table 6 show that listeners engage in a large number of unique multitasking activities, representing complex combinations of multi-sensory stimuli that push and pull for the listener's attention, alongside external stimuli perceptible in their surrounding environmental context. These findings support those presented by Baumgartner and Wiradhany (2021) that explored the shared modalities of media multitasking. The high variance observed amongst the different activities in these results would also support assertions made by Perks and Turner (2019) that podcast consumers engage in a wide variety of different multitasking activities, in part, to satisfy different divergent gratifications that can either command or release attentional resources and consequently engage an array of different attentional and cognitive processes in the listener.

Finally, the podcast discovery methods results shown in Table 8 indicate that while methods belonging to the podcasting ecosystem, namely podcast apps, podcasts themselves and other forms of online media, account for a majority of methods used (72.9%), listeners use a wide variety of different methods to discover podcasts, with over 25% of discovery methods being attributed to personal recommendation and offline media. These results highlight how portable listening devices such as smartphones and tablets are not only instrumental in podcast consumption, but also podcast discovery.

Collectively, the results of this present study strongly support those from prior studies that podcast consumption occurs across a wide variety of environmental (Chan-Olmsted and Wang, 2020), situational (Nyre, 2015), and social contexts (Perks and Turner, 2019).

#### 4.4.2. Media personalisation

The results of the EFA in the present study are valuable to the fields of personalized and object-based media, by investigating in which contexts listeners are most focused on the listening experience and, by extension, potentially more receptive to particular types of media personalisation. Understanding how podcast consumers' listening context might relate to their attentional engagement could also be valuable to producers looking to improve content personalisation and consequently maximize the revenue their podcasts are able to generate.

Research has identified an individual's participation with a podcast at the point of consumption, either directly by interacting with the creators on social media or indirectly by researching references made during the podcast, as a key factor in a listener's user-centered engagement (García-Marín, 2020). It also highlighted how the environmental, situational, and social context in which a podcast listening experience takes place can be a limiting factor in the listener's ability to engage in these forms of participatory engagement. For example, consuming podcasts while driving or engaging in multitasking activities that inhibit the listener's ability to physically interact with the listening device would severely limit affordances of participatory engagement.

Similarly, contextual limitations that inhibit affordances of participatory engagement, could also limit opportunities for listener engagement with various forms of explicit object-based personalisation that can be used to enhance the listening experience by various means, including improving audience accessibility (Ward et al., 2019), altering the length of media to suit listeners' time constraints (Armstrong et al., 2014), or creating interactive fictional stories that are responsive to user input (Ursu et al., 2020). In this sense, implicit forms of media personalisation, that automatically respond to elements of a user's context, facilitate the delivery of personalized media experiences across a much wider range of environmental and situational listening contexts, and as such are potentially better suited to the ubiquitous nature of podcast listening experiences highlighted by Morris and Patterson (2015) and the results of this present study.

#### 4.4.3. Attentional processing

The influence of bottom-up and top-down attention has been shown to extend far beyond auditory attention, with extensive research and debate relating to how bottom-up and top-down attention informs a wide array of multi-sensory and cognitive attentional processes (Hartcher-O'Brien et al., 2017). Results from this present study support assertions from existing research (Morris and Patterson, 2015) that podcasts are consumed at a variety of times, across different locations, under a range of environmental conditions, and often in parallel with a host of different multitasking activities. As such, podcast listening experiences engage a multitude of sensory and cognitive attentional processes, born of interactions with both the listener's environmental context and the podcast itself. It is therefore important to consider how the results of the present study might relate to bottom-up and top-down attention research.

The *soundscape & at work* factor of environmental context produced by the EFA in the present study can be analyzed in terms of the listener's environmental context, acting as either an active or passive influencing factor over their attentional engagement, both of which can potentially lead to higher attentional engagement by either focusing, or distracting and then focusing, the listener's attention.

If external stimuli in the listener's environment were sufficiently unobtrusive, such that it did not either positively or negatively capture their bottom-up attention, then they would best be placed to focus their goal orientated top-down attention on the podcast listening experience and better able to achieve higher attentional engagement. In this sense, the lack of sufficiently salient stimuli in the environment would passively enable the listener to experience higher attentional engagement.

In contrast, if stimuli in the listener's environment is sufficiently salient so as to negatively capture their bottom-up attention and be perceived as distracting or stress inducing, they may employ their goal orientated top-down attention in actively choosing to mask the external soundscape by listening to podcasts using a partially or fully occlusive headphone device. This would allow them to avoid the stresses of their environmental context by escaping into their own personal listening bubble. Consequently, they would be better placed to experience higher attentional engagement in the listening experience as a result of their active decision to listen to podcasts to counter the intrusive affects of their environmental context. This could also be considered an example of top-down attention being employed to lessen the sensitivity of bottom-up attention to otherwise highly salient stimuli in the environment, and as such is analogous to the shifting interactions between bottom-up and top-down attention, and auditory attention observed by Salmi et al. (2009), Bidet-Caulet et al. (2015), and Huang and Elhilali (2020).

Scenarios presented in this section have described ways in which a listener's bottom-up and top-down attentional processes can passively and actively interact with stimuli in their environment to facilitate higher attentional engagement in podcast listening experiences. However, as shown by the Likert response data in Figure 1, a significant proportion of participants also reported experiencing lower attentional engagement for the *soundscape & at work* items. Analysis of the free text qualitative responses indicates that some of the variance could be attributed to differences in the how favorably salient stimuli in the environment are perceived by the listener. It was revealed that some listeners preferred not to listen to podcasts while surrounded by natural stimuli because they preferred to focus their attention on the context of the natural environment and soundscape surrounding them. This suggests that salient environmental stimuli that captures an individual's bottom-up attention in a positive fashion, either because it is perceived as being sufficiently pleasurable or interesting, could lead to a wholesale loss in the listener's desire to focus their top-down directed attention toward engagement with a podcast listening experience.

These findings may be pertinent to research (White and Shah, 2019) in the field of attention restoration theory (Kaplan, 1995) that suggested the nature of the stimuli in an individual's surrounding physical environment can influence their attentional engagement and other aspects of cognitive performance. Urban environments, typically comprised of a high concentration of artificial man made stimuli, often tend to elicit forms of top-down directed attention in a way that can cause fatigue, potentially leading to a decrease in attentional engagement (Linnell et al., 2013). In contrast, natural environments that contain an abundance of natural stimuli are more likely to invoke automatic bottom-up attentional states that effortlessly capture the individual's attention and have the affect of replenishing individuals' cognitive control and capacity for attentional engagement (Berman et al., 2008). While it is important to recognize that the conclusions from these studies are not directly transferable to attentional engagement in podcast listening experiences, they do serve to illustrate how an individual's environmental context, and the nature of the stimuli contained within it, may influence their attentional engagement.

## 5. Conclusion

The results presented in this study are the first of their kind to support the hypothesis that a listener's environmental context exerts influence over their attentional engagement in podcast listening experiences. An online survey was used to collect data on the podcast listening engagement habits of a broad global sample. An EFA uncovered five *factors of environmental context* that both positively and negatively influence listeners' attentional engagement in podcast listening experiences. The *soundscape & at work* factor represented an especially insightful finding in its suggestion that podcast consumers actively choose to listen to podcasts as a form of soundscape masking. Separately, five *aspects of podcast listening engagement* were defined and measured across the sample, providing a comprehensive quantitative exploration of contemporary podcast listening experiences. Results together show that the proposed *factors of environmental context* are positively related to the *aspects of podcast listening engagement*, providing further validation and insight to the five defined *factors of environmental context*. Finally, the study considers how different forms of bottom-up and top-down attentional processing might relate to how environmental context influences attentional engagement in podcast listening, and the implications of the study for media personalisation.

Future work is required to provide further validation of the results in this present study, with acceptance of the results of the EFA being conditional on the findings of further confirmatory factor analysis studies. As the data for the present study was collected using a self-report survey, and largely based on participants' typical listening habits, it is also recommended that future studies should use a longitudinal study methodology, such as experience sampling method, to further improve the accuracy of results.

The findings are highly pertinent to the fields of podcast studies, media engagement, and mobile listening experiences. The results provide a basis for future research aiming to explore specific aspects of podcast listening engagement and factors of environmental context as they relate to episodes of listening in different environmental contexts. The research also has potential implications for future research exploring mobile audio listening and environmental context from the perspectives of media personalisation and attentional processing.

## Data availability statement

The original contributions presented in the study are included in the article/Supplementary material, further inquiries can be directed to the corresponding author.

## Ethics statement

The studies involving human participants were reviewed and approved by University of York Electronic Engineering Department Ethics Committee. The patients/participants provided their written informed consent to participate in this study.

## Author contributions

JH, DM, JF, and CP contributed to conception and design of the study. JH organized the database, performed the statistical analysis, and wrote the first draft of the manuscript. All authors contributed to manuscript revision, read, and approved the submitted version.
