# Supplementary material for: The relationship between environmental context and attentional engagement in podcast listening experiences
Source: Front Psychol. 2023 Jan 16;13:1074320. doi: 10.3389/fpsyg.2022.1074320 (PMC9885971; doi:10.3389/fpsyg.2022.1074320)
Supplement: Supplementary file 1 [file Data_Sheet_1.PDF]

Supplementary Figure 1. Survey Instrument and Survey Logic

Environmental Context Podcast Listening Habits Pilot Study

Start of Block: Participant Consent

Thank you for your interest in participating in this pilot study. Your contribution is highly valued and will aid research seeking to further understanding of how listening experiences are influenced by the environmental context in which they take place.

Instructions

In order to participate in this study you must have existing experience of listening to podcasts using a smartphone. The questionnaire is comprised of a series of multiple choice, ordinal scale & textual response questions and should take between 10 - 15 minutes to complete, depending on the extent of your listening experience.

Participant Consent

This questionnaire has been designed to adhere to the ethical codes and practices specified by the University of York. The personal data that is provided to this questionnaire by participants is anonymised and treated with the strictest confidentiality. Any elements of participant's personal data that enable identification will only be stored until the 31st of December 2021, after which it will be destroyed. Any anonymised personal data that is kept beyond this point will remain anonymised and will be used for the purposes of analysis and reporting results via internal and external publication.

In agreeing with this statement, you are confirming that you are over the age of 18, consenting to your participation in this questionnaire and the use of the anonymised personal data you provide in subsequent data analysis for the purposes of research conducted by the principal investigator. You are free to withdraw your consent for your participation in the study at any point.

This research is being conducted by the principal investigator, Jay Harrison (jah671@york.ac.uk) as part of an ongoing PhD project based in the AudioLab at the University of York. If you have any queries or wish to withdraw your consent from the study, please contact the principal investigator by email.

I confirm that I have read and understood the above and consent to my participation in the study.

○ Yes

○ No

End of Block: Participant Consent

Start of Block: Participation Qualification

Do you have experience of listening to podcasts using a smartphone?

○ Yes

○ No

End of Block: Participation Qualification

Start of Block: Personal Information

Display This Question:

If Do you have experience of listening to podcasts using a smartphone? = Yes

What is your age?

▼ 18-25 ... 66+

To which gender do you most identify?

- ◯ Male
- ◯ Female
- ◯ Non-binary / third gender
- ◯ Prefer not to say
- ◯ Other not listed (Please specify)

____

What is your country of residence?

____

What is your primary occupation?

- ◯ Employed (full time)
- ◯ Employed (part time)
- ◯ Unemployed
- ◯ Homemaker
- ◯ Student
- ◯ Retired
- ◯ None of the above

Display This Question:

If What is your primary occupation? = Employed (full time)

Or What is your primary occupation? = Employed (part time)

Or What is your primary occupation? = Student

When do you usually work?

- ◯ Weekdays (daytime)
- ◯ Weekdays (nights)
- ◯ Weekdays (alternating shifts)
- ◯ Weekends (daytime)
- ◯ Weekends (nights)
- ◯ Weekends (alternating shifts)
- ◯ Weekdays & weekends (daytime)
- ◯ Weekdays & weekends (nights)
- ◯ Weekdays & weekends (alternating shifts)
- ◯ Other not listed (Please specify) ____

Display This Question:

If What is your primary occupation? = Employed (full time)

Or What is your primary occupation? = Employed (part time)

Or What is your primary occupation? = Student

Do you currently commute to your occupation?

- ◯ Yes
- ◯ No

Display This Question:

If Do you currently commute to your occupation? = Yes

What method(s) of transportation do you usually use to commute to your occupation?

- ☐ Drive my own vehicle
- ☐ Passenger in someone else's vehicle
- ☐ Train
- ☐ Bus
- ☐ Bicycle
- ☐ Walk
- ☐ Other not listed (Please specify) ________________

End of Block: Personal Information

Start of Block: Podcast Information

In all of the questions in the next section of the survey, the term podcast is defined as any form of on-demand primarily speech based audio. This also includes on-demand radio programmes that are consumed after live transmission using a streaming service.

End of Block: Podcast Information

Start of Block: Playback p 1

What playback devices have you used with a smartphone to listen to podcasts? (Please select all that apply)

☐ Wired over-ear headphones

☐ Wired in-ear headphones

☐ Wireless over-ear headphones

☐ Wireless in-ear headphones

☐ Bluetooth speaker(s)

☐ Built-in smartphone speakers

☐ Built-in vehicle speakers

☐ Bone conduction headset

☐ Other not listed (Please specify) ____________________

End of Block: Playback p 1

Start of Block: Playback p 2

Display This Question:

If If What playback devices have you used with a smartphone to listen to podcasts? (Please select all that apply) q://QID39/SelectedChoicesCount Is Greater Than or Equal to 2

Carry Forward Selected Choices - Entered Text from "What playback devices have you used with a smartphone to listen to podcasts? (Please select all that apply)"

X→

Which of the following playback devices do you most often use with a smartphone to listen to podcasts? (Please select one)

- ○ Wired over-ear headphones
- ○ In-ear headphones
- ○ Wireless over-ear headphones
- ○ Bluetooth earbuds (i.e. Airpods
- ○ Bluetooth speaker(s)
- ○ Built-in smartphone speakers
- ○ Built-in vehicle speakers
- ○ Bone conduction headset
- ○ Other not listed (Please specify)

End of Block: Playback p 2

Start of Block: Weekday p

On an average **weekday**, how much time do you typically spend listening to podcasts on a smartphone?

▼ 0 minutes ... More than 10 hours

What percentage of this time is typically spent listening whilst simultaneously engaging in other activities?

▼ 0% (I never engage in other activities whilst listening on weekdays) ... 100% (I always engage in other activities whilst listening on weekdays)

End of Block: Weekday p

Start of Block: Weekend p

On an average **weekend day**, how much time do you typically spend listening to podcasts on a smartphone?

▼ 0 minutes ... More than 10 hours

What percentage of this time is typically spent listening whilst simultaneously engaging in other activities?

▼ 0% (I never engage in other activities whilst listening on weekend days) ... 100% (I always engage in other activities whilst listening on weekend days)

End of Block: Weekend p

Start of Block: Multitasking p 1

Display This Question:

If What percentage of this time is typically spent listening whilst simultaneously engaging in other... != 0% (I never engage in other activities whilst listening on weekdays)

Or What percentage of this time is typically spent listening whilst simultaneously engaging in other... != 0% (I never engage in other activities whilst listening on weekend days)

What activities have you also engaged in while simultaneously listening to podcasts using a smartphone? (Please select all that apply)

- ☐ Watching TV
- ☐ Reading
- ☐ Listening to music
- ☐ Talking on the phone
- ☐ Sending messages via phone or computer
- ☐ Using social media sites
- ☐ Watching films
- ☐ Other computer activities
- ☐ Playing video games
- ☐ Doing housework
- ☐ Preparing food
- ☐ Exercising
- ☐ Other not listed (Please specify) ____

End of Block: Multitasking p 1

Start of Block: Multitasking p 2

Display This Question:

If If What activities have you also engaged in while simultaneously listening to podcasts using a smartphone? (Please select all that apply) q://QID45/SelectedChoicesCount Is Greater Than or Equal to 2

Carry Forward Selected Choices - Entered Text from "What activities have you also engaged in while simultaneously listening to podcasts using a smartphone? (Please select all that apply)"

X→

Which of the following activities do you most often also engage in while simultaneously listening to podcasts using a smartphone? (Please select one)

- ○ Watching TV
- ○ Reading
- ○ Listening to music
- ○ Talking on the phone
- ○ Sending messages via phone or computer
- ○ Using social media sites
- ○ Watching films
- ○ Other computer activities
- ○ Playing video games
- ○ Doing housework
- ○ Preparing food
- ○ Exercising
- ○ Other not listed (Please specify)

End of Block: Multitasking p 2

Start of Block: Location p 1

What locations have you listened to podcasts in using a smartphone? (Please select all that apply)

- ☐ At home
- ☐ At work
- ☐ Driving a vehicle
- ☐ Traveling on public transport
- ☐ On a run
- ☐ At the gym
- ☐ Walking in a rural environment
- ☐ Walking in an urban environment
- ☐ Other not listed (Please specify) ____

End of Block: Location p 1

Start of Block: Location p 2

Display This Question:

If If What locations have you listened to podcasts in using a smartphone? (Please select all that apply) q://QID47/SelectedChoicesCount Is Greater Than or Equal to 2

Carry Forward Selected Choices - Entered Text from "What locations have you listened to podcasts in using a smartphone? (Please select all that apply)"

X→

In which of the following locations do you most often listen to podcasts using a smartphone? (Please select one)

- ○ At home
- ○ At work
- ○ Driving a vehicle
- ○ Traveling on public transport
- ○ On a run
- ○ At the gym
- ○ Walking in a rural environment
- ○ Walking in an urban environment
- ○ Other not listed (Please specify)

End of Block: Location p 2

Start of Block: Discovery p g 1

What methods have you used to discover podcasts? (Please select all that apply)

- ☐ Streaming services (i.e. Spotify, Pandora, etc.)
- ☐ Searching the internet
- ☐ Listening to podcasts
- ☐ Listening to radio
- ☐ Recommendations from friends/family
- ☐ Recommendations on social media
- ☐ Recommendations from YouTube creators
- ☐ Recommendations from YouTube based on your viewing history
- ☐ Other not listed (Please specify) ____

End of Block: Discovery p g 1

Start of Block: Discovery p g 2

Display This Question:

If If What methods have you used to discover podcasts? (Please select all that apply) q://QID49/SelectedChoicesCount Is Greater Than or Equal to 2

Carry Forward Selected Choices - Entered Text from "What methods have you used to discover podcasts? (Please select all that apply)"

X→

Which of the following methods do you most often use to discover podcasts? (Please select one)

- ◯ Recommendations from streaming services (i.e. spotify, pandora, etc.)
- ◯ Searching the internet
- ◯ Curated recommendations from audio based media (i.e. podcasts, radio, etc.)
- ◯ Adverts on social media
- ◯ Personal Recommendations from friends/family
- ◯ Recommendations on social media
- ◯ Recommendations from YouTube creators
- ◯ Recommendations from YouTube based on your viewing history
- ◯ Other not listed (Please specify)

End of Block: Discovery p g 2

Start of Block: Discovery p s 1

Display This Question:

If What methods have you used to discover podcasts? (Please select all that apply) = Streaming services (i.e. Spotify, Pandora, etc.)

What methods have you used to discover podcasts on streaming services? (Please select all that apply)

▢ Using the search bar and/or browsing to make self directed selections

▢ Personally curated playlists and/or recommendations that follow a particular theme

▢ Playlisting and/or recommendations generated from my listening history

▢ General editorial recommendations/adverts not related to my listening history or any particular theme

▢ Other not listed (Please specify) ____

End of Block: Discovery p s 1

Start of Block: Discovery p s 2

Display This Question:

If If What methods have you used to discover podcasts on streaming services? (Please select all that apply) q://QID51/SelectedChoicesCount Is Greater Than or Equal to 2

Carry Forward Selected Choices - Entered Text from "What methods have you used to discover podcasts on streaming services? (Please select all that apply)"

X→

Which of the following methods do you most often use to discover podcasts on streaming services? (Please select one)

○ Using the search bar and/or browsing to make self directed selections

○ Personally curated playlists and/or recommendations that follow a particular theme

○ Playlisting and/or recommendations generated from my listening history

○ General editorial recommendations/adverts not related to my listening history or any particular theme

○ Other not listed (Please specify)

End of Block: Discovery p s 2

Start of Block: Subscription p

Do you currently make a regular financial contribution to consume podcasts or support podcast creators (i.e. Spotify or Patreon)? (Please select one)

○ Yes, I pay to consume podcasts on a streaming service (i.e. Spotify)

○ Yes, I pay to support podcast creators on a subscription service (i.e. Patreon)

○ Yes, I pay to consume podcasts on a streaming service (i.e. Spotify) & also pay to support podcast creators on a subscription service (i.e. Patreon)

○ No

End of Block: Subscription p

Start of Block: Skipping p

How much of a podcast do you typically listen to on a smartphone before searching for something else that better satisfies your listening preferences?

▼ Less than 5% ... Not applicable

End of Block: Skipping p

Start of Block: Attention p 1

How would you describe the attention you typically afford to the **listening experience** when consuming **podcasts on a smartphone**? (Please select one)

- ○ Focused
- ○ Sustained
- ○ Selective
- ○ Alternating
- ○ Divided
- ○ Background
- ○ Other not listed (Please specify) ____

End of Block: Attention p 1

Start of Block: Attention p 2

How would you describe the attention you typically afford to the **listening experience** when consuming **podcasts on a smartphone** as the **only activity occupying your attention**? (Please select one)

- ○ Focused
- ○ Sustained
- ○ Selective
- ○ Alternating
- ○ Divided
- ○ Background
- ○ Other not listed (Please specify) ____
- ○ Not applicable

End of Block: Attention p 2

Start of Block: Attention p 3

How would you describe the attention you typically afford to the **listening experience** when consuming **podcasts on a smartphone** and **simultaneously engaging in other activities**? (Please select one)

- ○ Focused
- ○ Sustained
- ○ Selective
- ○ Alternating
- ○ Divided
- ○ Background
- ○ Other not listed (Please specify) ____
- ○ Not applicable

End of Block: Attention p 3

Start of Block: Attention p 4

How would you describe the attention you typically afford to **your surroundings** when listening to **podcasts on a smartphone**? (Please select one)

- ○ Focused
- ○ Sustained
- ○ Selective
- ○ Alternating
- ○ Divided
- ○ Background
- ○ Other not listed (Please specify) ____

End of Block: Attention p 4

Start of Block: Actively Engaged Information

In the following section of the survey the term 'actively engaged' is used to describe an attentional state where the listener is fully focused on the listening experience.

End of Block: Actively Engaged Information

Start of Block: Podcasts Time EFA

I feel actively engaged in the listening experience when using a smartphone to consume podcasts...

	Likert Scale					
	Strongly disagree	Disagree	Neither agree nor disagree	Agree	Strongly agree	Not applicable

on weekdays in the morning (08:00-08:59)	◯	◯	◯	◯	◯	◯
on weekdays during the daytime (09:00-16:59)	◯	◯	◯	◯	◯	◯
on weekdays in the early evening (17:00-20:59)	◯	◯	◯	◯	◯	◯
on weekdays in the late evening (21:00-23:59)	◯	◯	◯	◯	◯	◯
on weekend days in the morning (08:00-08:59)	◯	◯	◯	◯	◯	◯
on weekend days during the daytime (09:00-16:59)	◯	◯	◯	◯	◯	◯
on weekend days in the early evening (17:00-20:59)	◯	◯	◯	◯	◯	◯
on weekend days In the	◯	◯	◯	◯	◯	◯

late evening (21:00-23:59)

End of Block: Podcasts Time EFA

Start of Block: Podcasts Location EFA

I feel actively engaged in the listening experience when using a smartphone to consume podcasts...

	Likert Scale					
	Strongly disagree	Disagree	Neither agree nor disagree	Agree	Strongly agree	Not applicable
at home	○	○	○	○	○	○
at work	○	○	○	○	○	○
whilst driving a vehicle	○	○	○	○	○	○
on public transport	○	○	○	○	○	○
on a run	○	○	○	○	○	○
at the gym	○	○	○	○	○	○
on a walk in a rural environment	○	○	○	○	○	○
on a walk in an urban environment	○	○	○	○	○	○

End of Block: Podcasts Location EFA

Start of Block: Podcasts Sound EFA

I feel actively engaged in the listening experience when using a smartphone to consume podcasts...

	Likert Scale					
	Strongly disagree	Disagree	Neither agree nor disagree	Agree	Strongly agree	Not applicable

in an environment with low levels of background noise	○	○	○	○	○	○
in an environment with moderate levels of background noise	○	○	○	○	○	○
in an environment with high levels of background noise	○	○	○	○	○	○
in an environment mainly comprised of sounds that indicate the presence of humans (i.e. speech, footsteps, music & laughter)	○	○	○	○	○	○
in an environment mainly comprised of sounds that indicate natural activity not related to humans (i.e. birdsong, animal calls, running water, wind & ocean waves)	○	○	○	○	○	○

in an environment mainly comprised of mechanical sounds (i.e. machinery, transport & construction)	○	○	○	○	○	○

End of Block: Podcasts Sound EFA

Start of Block: Podcasts Weather EFA

I feel actively engaged in the listening experience when using a smartphone to consume podcasts...

	Likert Scale					
	Strongly disagree	Disagree	Neither agree nor disagree	Agree	Strongly agree	Not applicable

outdoors in warm conditions	○	○	○	○	○	○
outdoors in cold conditions	○	○	○	○	○	○
outdoors in light conditions	○	○	○	○	○	○
outdoors in dark conditions	○	○	○	○	○	○
indoors in warm conditions	○	○	○	○	○	○
indoors in cold conditions	○	○	○	○	○	○
indoors in light conditions	○	○	○	○	○	○
indoors in dark conditions	○	○	○	○	○	○

End of Block: Podcasts Weather EFA

Start of Block: Thematic p

Please enter any observations you may have on how different factors of environmental context influence your engagement level when listening to podcasts using a smartphone:

____

Please enter any observations you may have on how different factors of environmental context influence your preference for listening to specific types of podcasts using a smartphone:

____

End of Block: Thematic p
